# Topological control of the *Caulobacter* cell cycle circuitry by a polarized single-domain PAS protein

**DOI:** 10.1038/ncomms8005

**Published:** 2015-05-08

**Authors:** Stefano Sanselicio, Matthieu Bergé, Laurence Théraulaz, Sunish Kumar Radhakrishnan, Patrick H. Viollier

**Affiliations:** 1 Department of Microbiology and Molecular Medicine, Institute of Genetics and Genomics in Geneva (iGE3), Faculty of Medicine/CMU, University of Geneva, Rue Michel Servet 1, Genève 4 1211, Switzerland

## Abstract

Despite the myriad of different sensory domains encoded in bacteria, only a few types are known to control the cell cycle. Here we use a forward genetic screen for *Caulobacter crescentus* motility mutants to identify a conserved single-domain PAS (Per-Arnt-Sim) protein (MopJ) with pleiotropic regulatory functions. MopJ promotes re-accumulation of the master cell cycle regulator CtrA after its proteolytic destruction is triggered by the DivJ kinase at the G1-S transition. MopJ and CtrA syntheses are coordinately induced in S-phase, followed by the sequestration of MopJ to cell poles in *Caulobacter*. Polarization requires *Caulobacter* DivJ and the PopZ polar organizer. MopJ interacts with DivJ and influences the localization and activity of downstream cell cycle effectors. Because MopJ abundance is upregulated in stationary phase and by the alarmone (p)ppGpp, conserved systemic signals acting on the cell cycle and growth phase control are genetically integrated through this conserved single PAS-domain protein.

## Abstract (web summary)

The bacterium *Caulobacter crescentus* is a model organism for research on the bacterial cell cycle and cell division processes. Here, Sanselicio *et al*. show that the MopJ protein contributes to the control of cell cycle and growth in *C. crescentus.*

## Graphical abstract

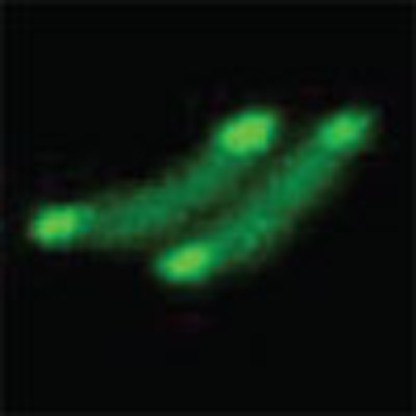

Cellular motility is responsive to external signals such as nutritional changes, but it is also regulated by cues induced systemically during each cell division cycle^1^,^2^. This latter characteristic has been successfully exploited in motility screens to uncover cell cycle regulators in the model bacterium *Caulobacter crescentus* (herein *Caulobacter*), an aquatic α-Proteobacterium that is easily synchronized by density-gradient centrifugation owing to a cell cycle-regulated capsule^3^,^4^,^5^,^6^. Motility is conferred by the polar flagellum, a structure that is required for the dispersal of the swarmer cell type. *Caulobacter* divides asymmetrically into a swarmer cell that resides in a G1-like quiescent state and harbours the flagellum and several pili at the same cell pole, and a replicative (S-phase) cell whose old cell pole is decorated by a cylindrical extension of the cell envelope (the stalk) tipped by an adhesive holdfast (Fig. 1a)^2^. Flagellar motility along with adhesive properties (conferred by the polar pili and holdfast) and cell division are wired into the *C. crescentus* cell cycle circuitry at the transcriptional level by the master cell cycle regulator CtrA^7^, a DNA-binding response regulator (RR) of the OmpR family^4^ whose synthesis is activated in S-phase by the transcriptional regulator GcrA^8^. Following its synthesis, CtrA activates many developmentally regulated promoters, including those of motility, pilus, holdfast and cell division genes^7^,^9^,^10^,^11^. CtrA also functions as negative regulator of gene expression and, directly and/or indirectly, inhibits firing of the origin of DNA replication (*Cori*). *Cori* fires only once during the *Caulobacter* cell cycle and is bound by CtrA at multiple sites^12^,^13^.

The DNA-binding activity of RRs such as CtrA is regulated by phosphorylation at a conserved (Asp) residue, a step that is often executed directly by a histidine kinase (HK)^7^. Before the phosphate can be accepted by the RRs, the dimeric HK *trans*-auto-phosphorylates a conserved histidine (His) in an ATP-dependent manner. However, phosphorylation of RRs can also occur indirectly by way of two intermediary components in the His-Asp phosphorylation pathway, an Asp-containing receiver domain and a His-containing phosphor-transfer domain^14^. Complex regulatory schemes emerge when such multi-component His-Asp pathways are arranged in tandem^15^. The accumulation of phosphorylated CtrA (CtrA∼P) underlies such a complex His-Asp pathway topology^15^,^16^,^17^. CtrA∼P is present in G1-phase, degraded at the G1-S transition by the ClpXP protease^18^,^19^, to enable the initiation of DNA replication, and then re-synthesized and phosphorylated during S-phase (Fig. 1a). The removal of CtrA∼P at the G1-S transition is induced when the DivJ HK phosphorylates its substrate, the DivK receiver domain, which downregulates both the phosphorylation and half-life of CtrA via the DivL tyrosine kinase^15^,^16^,^20^,^21^,^22^,^23^,^24^. CtrA abundance and stability are also regulated by the conserved alarmone (p)ppGpp, which tunes cell cycle progression in response to nutritional signals^25^,^26^,^27^,^28^,^29^.

Superimposed on the temporal events is the spatial regulation of these His-Asp pathway components (Fig. 1a)^30^. At the G1-S transition, DivJ is recruited to the stalked pole by its localization factor SpmX, an event that also is required for optimal DivJ kinase activity *in vivo*^3^,^31^,^32^. DivK is enriched at the poles as well, enhancing kinase activity^33^, but a significant fraction of DivK is also dispersed^17^,^34^. DivK activity is antagonized by the phosphatase PleC that is recruited to the pole opposite the stalk by its localization factor PodJ^32^,^35^,^36^,^37^. A pleiotropic effector of protein polarization in *C. crescentus* is the polar organizing protein Z that is thought to self-assemble into a polar matrix at both cell poles to sequester proteins like SpmX, DivJ, DivK and others^38^,^39^,^40^,^41^. Although the DivJ and SpmX complex is unipolar, DivK localization is bipolar and promoted by SpmX and DivJ activity^17^,^38^, indicating that DivK localization opposite the stalk is regulated by another factor. The sequestration of DivK to the stalked pole is governed by DivJ, even in the absence of kinase activity^17^.

Here, we report a screen for motility mutants that unearthed a conserved and cell cycle-regulated single-domain PAS (Per-Arnt-Sim)^42^ protein (MopJ) that promotes CtrA accumulation during exponential growth and in stationary phase. MopJ is localized to the cell poles where it acts on downstream cell cycle signalling proteins, whereas upstream cell cycle regulators promote MopJ polarization. Our work unveils MopJ as a modulator of the spatio-temporal circuitry controlling bacterial cell cycle progression and as a regulatory node through which stationary phase and (p)ppGpp-dependent nutritional signals are integrated.

## Results

### MopJ is a pleiotropic regulator of motility

**Figure 1:**
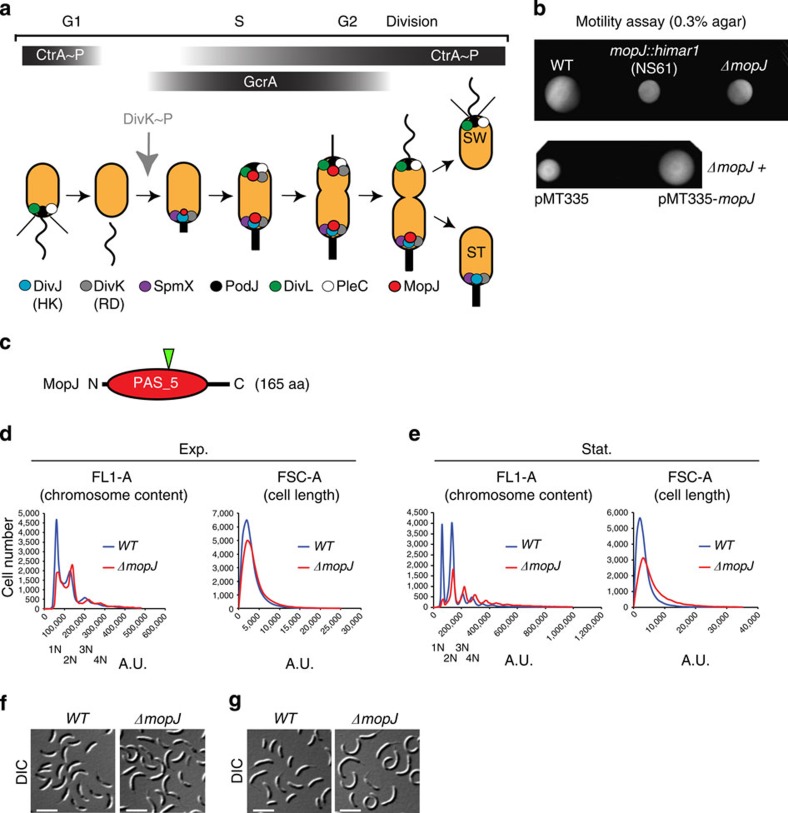
MopJ, a pleiotropic regulator controlling motility and cell cycle progression in *Caulobacter crescentus*. (**a**) Model showing the localization of His-Asp signalling proteins and its regulators along with MopJ during *Caulobacter crescentus* cell cycle. The black bars show the abundance of the transcriptional regulators CtrA (and phosphorylated CtrA, CtrA∼P) and GcrA. The grey arrow indicates when phosphorylation of the DivK receiver domain (RD) by the DivJ histidine kinase (HK) commences. Phosphorylated DivK (DivK∼P) is present henceforth. The thin vertical line in black represents the flagellum before it rotates (wavy line). The thick vertical line in black represents the stalk. The thin slanted black lines represent the polar pili. (**b**) (Upper panel) Motility assay on swarm agar plate of *WT*, *mopJ::himar* and Δ*mopJ* cells. (Lower panel) complementation of Δ*mopJ* cells harbouring the empty vector pMT335 or pMT335-*mopJ.* (**c**) Scheme of domain organization of MopJ from N- to C-terminus indicated by the total amino-acid (aa) length. The green arrowhead points to the residue in the *mopJ* coding sequence that was disrupted by the *himar1* transposon insertion. (**d**,**e**) FACS analysis of *WT* and Δ*mopJ* cells. Genome content (FL1-A channel) and cell size (FSC-A channel) were analysed by FACS during exponential (**d**, Exp.) and stationary (**e**, Stat.) phases in M2G. G1 (1 N) and G2 cell (2 N) peaks of fluorescence intensity are readily visible (particularly in Stat. phase cells) and are labelled accordingly. S-phase cells are reflected by the broad intermittent signal (absent in Stat. phase cells). (**f**,**g**) Differential interference contrast (DIC) images of *WT* and Δ*mopJ* cells during Exp. (**f**) and Stat. (**g**) phases in M2G.

Our previous comprehensive transposon mutagenesis screen for strains with a motility defect on swarm (0.3%) agar that led to the identification of the *spmX* gene^3^ also yielded one mutant strain (NS61, Fig. 1b) harbouring a *himar1* insertion in the uncharacterized gene *CCNA_00999* (at nucleotide position 1082252 of the *Caulobacter crescentus* wild-type (*WT*) strain NA1000 (ref. 43). This gene is predicted to encode a single-domain PAS protein (PAS_5, pfam07310, residues 15–147, Fig. 1c) that is 165 residues in length and is henceforth referred to as MopJ (motility PAS domain associated with DivJ, see below). The PAS domain, a five-stranded antiparallel β-sheet flanked by several α-helices, is generally used to bind small-molecule ligands and is structurally related to the GAF regulatory (GMP-specific and -regulated cyclic nucleotide phosphodiesterase, Adenylyl cyclase and *Escherichia coli* transcription factor FhlA) domain often encoded on the same polypeptide^42^. Orthologues of MopJ are also encoded in the genomes of distantly related α-Proteobacteria (Supplementary Fig. 1) such as the animal pathogen *Brucella melitensis* (BMEI0738), the plant pathogen *Agrobacterium tumefaciens* (Atu1754) and the plant symbiont *Sinorhizobium meliloti* (SMc01000). These proteins exhibit a similar domain organization with MopJ, that is, a single PAS_5 domain without any associated regulatory or effector domain (Fig. 1c). The *himar1* transposon in NS61 lies near the middle of the *mopJ* gene (that is, after codon 89, Fig. 1c), presumably disrupting its function. In support of this notion, an in-frame deletion of *mopJ* (Δ*mopJ*) in *WT* cells recapitulated the motility defect observed in the *mopJ*::*himar1* mutant on soft agar (Fig. 1b). As the motility defect of Δ*mopJ* cells can be corrected by supplying *mopJ in trans* on a plasmid (pMT335-*mopJ*) under the control of the vanillate-inducible promoter P_*van*_ (Supplementary Fig. 2a), we conclude that MopJ is a hitherto uncharacterized regulator of motility in *C. crescentus*.

As inactivation of cell cycle regulators often results in pleiotropic defects in *C. crescentus* including a reduction of motility^5^ caused by a change in the fraction of G1-phase cells in the population, we probed for such changes by fluorescence-activated cell sorting (FACS) analysis of exponentially growing *WT* and Δ*mopJ* cells stained with the nucleic acid dye SYTOX Green (Fig. 1d). A lower G1:G2 cell ratio was observed in Δ*mopJ* cultures compared with *WT*, while the number of S-phase cells seemed unaffected. By contrast, stationary *WT* or Δ*mopJ* cultures contained few S-phase cells, confirming that the majority of cells are no longer replicating (Fig. 1e). Although stationary *WT* cells have an equal tendency to arrest either in G1- or G2-phase, stationary Δ*mopJ* cells are enriched in G2-phase over G1-phase. Consistent with these findings, differential interference contrast (DIC) microscopy and FACS revealed only slight increase in cell length caused by the Δ*mopJ* mutation in exponential phase (Fig. 1d,f), whereas in stationary phase many Δ*mopJ* cells are elongated and appear to divide aberrantly (Fig. 1e,g).

Taken together, our results reveal MopJ as a novel pleiotropic regulator of motility and of normal cell cycle progression in *C. crescentus.* Specifically, MopJ promotes the accumulation of G1-phase cells in exponential phase and of G1- and G2-phase cells in stationary phase.

### MopJ acts on the CtrA regulon and is induced by (p)ppGpp

**Figure 2:**
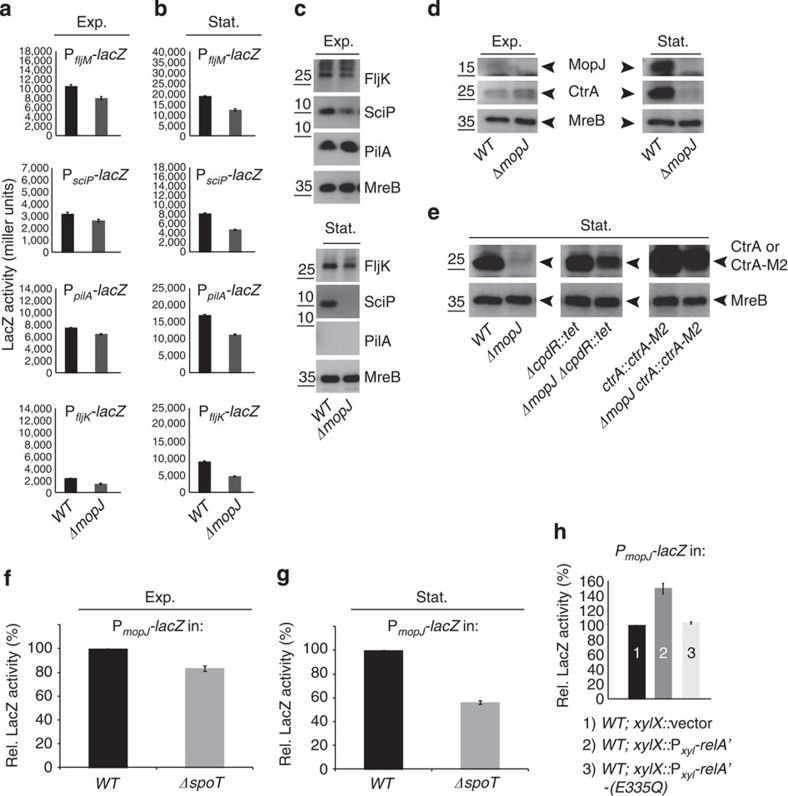
MopJ promotes CtrA accumulation and is regulated by (p)ppGpp. (**a**,**b**) Promoter-probe assays of transcriptional reporters carrying the *fljM*, *sciP*, *pilA* or *fljK* promoter fused to a promoterless *lacZ gene* in *WT* and Δ*mopJ* cells in exponential (**a**, Exp.) and stationary (**b**, Stat.) phase. The graphs show *lacZ*-encoded β-galactosidase activities measured in Miller Units. (**c**) Immunoblot showing the steady-state levels of the major flagellin FljK, the SciP-negative regulator and the PilA structural subunit of the pilus filament in *WT* and Δ*mopJ* cells in Exp. and Stat. phase. The steady-state levels of the MreB actin are shown as a loading control (see Supplementary Fig. 4A). (**d**) Immunoblots showing CtrA and MopJ abundance in Exp. and Stat. phase Δ*mopJ versus WT* cells. Steady-state levels of MreB are shown as loading control (see Supplementary Fig. 4B). (**e**) Accumulation of CtrA is increased in Δ*mopJ* cells during Stat. phase either by inactivation of a proteolytic regulator encoded by *cpdR* (Δ*cpdR*::*tet)* or by appending an M2 (FLAG) epitope to the C-terminus of CtrA (*ctrA*::*ctrA-M2*). Immunoblot showing the steady-state levels of CtrA in *WT*, Δ*mopJ*, Δ*cpdR*::tet and Δ*mopJ* Δ*cpdR*::*tet* cells and the levels of CtrA-M2 in *ctrA*::*ctrA-M2* and Δ*mopJ ctrA*::*ctrA-M2* cells. Steady-state levels of MreB are shown as loading control. (**f**,**g**) Promoter-probe assays of transcriptional reporters carrying the P_*mopJ*_-*lacZ* in *WT* and Δ*spoT* cells in Exp. (**f**) and Stat. (**g**) phase. (**h**) LacZ assays with P_*mopJ*_-*lacZ* promoter-probe plasmid in *WT* and cells expressing the constitutive active form of *E. coli* RelA (RelA') and the catalytic mutant (RelA'-E335Q) fused to the FLAG tag in the presence of xylose. (**a**,**b**,**f**,**g**,**h**) Error bar (black bar) is shown as standard deviation (s.d., *n*=3).

Knowing that MopJ impacts both motility and the abundance of G1 cells, we tested if MopJ affects the expression of the CtrA regulon, as this includes motility, division and G1-phase genes^9^,^10^,^12^. In addition, this master transcriptional regulator is a likely target of MopJ because it also regulates the initiation of chromosome replication^4^,^12^. To this end, we measured if β-galactosidase (LacZ) expression from CrA-regulated promoters is altered in exponential and stationary phase *WT* and Δ*mopJ* cells harbouring P_*pilA*_-, P_*sciP*_- or P_*fljM*_-*lacZ* promoter probe plasmids (harbouring LacZ under the control of the promoter of the *pilA* pilin gene, the *sciP* repressor gene or the *fljM* minor flagellin gene, Fig. 2a,b) that are directly activated by CtrA^9^,^10^,^44^ versus a *podJ* promoter reporter (Supplementary Fig. 3A) that is repressed by CtrA. Although only a modest reduction in LacZ activity (14–25%, depending on the CtrA-activated promoter, see Supplementary Table 1) was discernible in exponentially growing Δ*mopJ* cells compared with *WT* cells (Fig. 2a), this difference was accentuated in stationary phase (Fig. 2b and Supplementary Table 1) and even further magnified in a reporter that is indirectly activated by CtrA such as P_*fljK*_-*lacZ*, in which the promoter of the major flagellin gene *fljK* drives LacZ expression (Fig. 2a,b and Supplementary Table 1). P_*fljK*_ is a target of the FlbD activator whose expression in turn is directly and positively regulated by CtrA^9^,^10^.

Immunoblotting using polyclonal antibodies to FljK, SciP and PilA confirmed the trends observed with the LacZ transcriptional reporters (Fig. 2c and Supplementary Fig. 4a). (Note that the PilA protein is absent from stationary phase *WT* cells for reasons that are currently unknown, but is likely operating at the post-transcriptional level (compare Fig. 2b,c and Supplementary Fig. 4a). Immunoblotting using antibodies to CtrA revealed that CtrA abundance is strongly dependent on MopJ in stationary phase (Fig. 2d and Supplementary Fig. 4b), consistent with the exacerbated effects on CtrA and its regulon in stationary Δ*mopJ* cells compared with *WT* cells. Stabilizing CtrA either by masking the C-terminal recognition motifs for ClpXP in CtrA (using the *ctrA*::*ctrA*-*M2* allele^45^) or by inactivating the gene encoding the CpdR proteolytic activator of CtrA^16^,^20^ restores CtrA abundance to near *WT* levels (Fig. 2e), indicating that CtrA proteolysis by the ClpXP protease contributes to downregulation of CtrA levels in stationary Δ*mopJ* cells.

**Figure 3:**
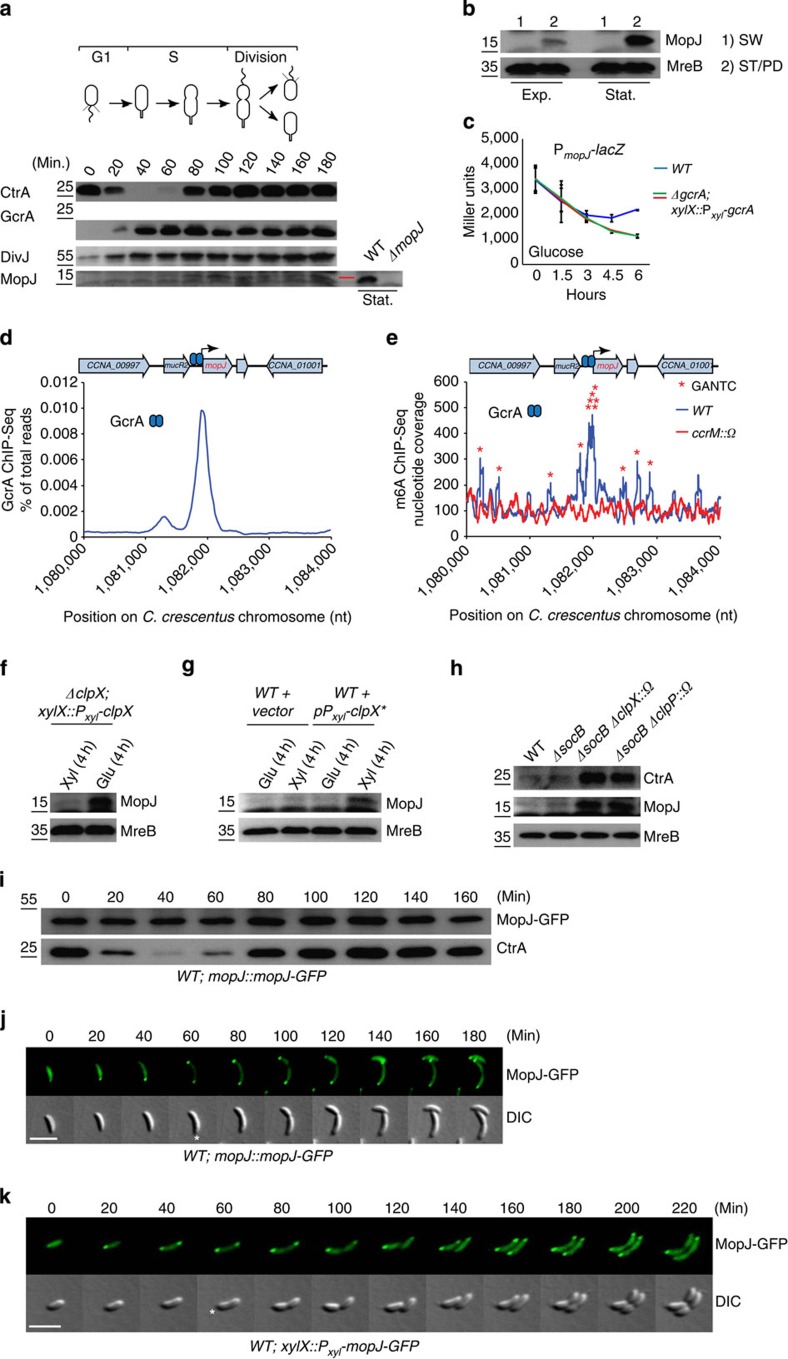
Multi-layered spatiotemporal regulation of MopJ. (**a**) Immunoblots showing steady-state levels of MopJ, GcrA, CtrA and DivJ in synchronized *WT* cells and MopJ in stationary (Stat.) phase (**a**, right, see Supplementary Fig. 5a,b). The red line marks the position of MopJ. (**b**) MopJ levels in exponential (Exp.) or stationary (Stat.) phase swarmer (SW) and stalked (ST)/predivisional (PD) cells detected using antibodies to MopJ (see Supplementary Fig. 5c). (**c**) LacZ measurements (Miller units) of P_*mopJ*_-*lacZ* in *WT* (blue) and two independent Δ*gcrA xylX*::P_*xyl*_*-gcrA* (red and green) mutants, during 6 h (hours) of inhibition (glucose) of *gcrA* expression. Note the decline in activity upon dilution of overnight cells is due to the prior induction in stationary phase by (p)ppGpp (see Fig. 2f–h and Supplementary Fig. 3b). Error bar (black bar) is shown as s.d. (*n*=3). (**d**) Occupancy of GcrA (blue ovals) at the *mopJ* locus in *WT* cells as determined by chromatin immunoprecipitation coupled to deep-sequencing (ChIP-Seq) using polyclonal antibodies to GcrA^50^. Occupancy is shown as percentage of total reads in the precipitated sample as a function of the chromosome coordinates. (**e**) Trace of N6-methyladenosine (m6A) marked DNA at *mopJ* locus in *WT* cells (blue trace) and Δ*ccrM::*Ω (red trace) from ChIP-Seq experiment performed with antibodies to m6A^50^. Occupancy is shown as percentage of total nucleotide coverage in sequencing as a function of the chromosome coordinates at the *mopJ* locus. Stars show the positions of 5′-GANTC-3′ sequences that are methylated by the CcrM methyltransferase at the N6 position of adenine. (**f**–**h**) Immunoblots showing the steady-state levels of MopJ in Δ*clpX xylX*::P*xyl-clpX* cells with (Xyl, xylose) or without (Glu, glucose) induction of P_*xyl*_ for 4 h (**f**), upon induction of the dominant negative form ClpX* from P_*xyl*_ for 4 h (**g**), and in *WT*, Δ*socB* and Δ*socB*Δ*clpX*::Ω, Δ*socB*Δ*clpP*::Ω double mutants (**h**) along with CtrA and MreB (control). (**i**) Immunoblot showing MopJ-GFP and CtrA levels in synchronized *mopJ::mopJ-GFP* cells (see Supplementary Fig. 5d). (**j**,**k**) Time-lapse fluorescence imaging of live *mopJ*::*mopJ-GFP* cells (**j**) or *WT xylX*::P_*xyl*_*-mopJ-GFP* cells (**k**). White asterisk denotes stalked pole. (**a**,**i**,**j**,**k**) Numbers indicate minutes after synchronization.

Next, we raised antibodies to MopJ to probe for commensurate changes in MopJ abundance in stationary phase versus exponential phase. In support of the stationary phase defects of Δ*mopJ* cells described above, we observed that MopJ was barely detectable in lysates from exponential phase cells, but is abundant in lysates from stationary phase cells (Fig. 2d and see Fig. 3a and Supplementary Fig. 4b). As the alarmone (p)ppGpp accumulates in stationary phase when nutrients become exhausted in bacteria^25^,^46^, we asked if the stationary phase induction of MopJ requires the (p)ppGpp-synthase/hydrolase SpoT^27^,^28^,^47^. To resolve this question, we conducted β-galactosidase (LacZ) measurements in cells harbouring a transcriptional reporter, in which a promoterless *lacZ* gene is fused to the *mopJ* promoter (P_*mopJ*_-*lacZ*). Accordingly, we measured P_*mopJ*_-*lacZ* activity during exponential and stationary phase in the presence or absence of SpoT (that is, in *WT* and Δ*spoT* cells, respectively), observing a strong (50%) reduction of P_*mopJ*_-*lacZ* expression in stationary cells lacking SpoT (Fig. 2f,g). To demonstrate that (p)ppGpp induction is sufficient for the P_*mopJ*_-*lacZ* induction even in exponential phase cells, we induced (p)ppGpp synthesis in exponential phase cells using the constitutively active (p)ppGpp synthase RelA' from *E. coli*^47^ and detected a commensurate increase in P_*mopJ*_-*lacZ* activity (Fig. 2h). By contrast, no induction was observed upon induction of the catalytic mutant derivative RelA'-E335Q (Fig. 2h), demonstrating that (p)ppGpp is necessary and sufficient for induction of P_*mopJ*_-*lacZ.*

Thus, MopJ acts on CtrA and its regulon, especially in stationary phase, and (p)ppGpp signalling by SpoT induces MopJ at the transcriptional level in stationary phase cells.

### Coordinated synthesis of MopJ and CtrA in S-phase

The functional relationship between MopJ and CtrA is further reinforced by their concurrent accumulation during the cell cycle, coordinated by the S-phase regulator GcrA. Immunoblotting revealed that MopJ appears during the cell cycle coincident with or slightly ahead of CtrA and follows the accumulation of GcrA and DivJ (Fig. 3a and Supplementary Fig. 5a,b). Separation of exponential and stationary phase *WT* cells into swarmer (G1-phase) and stalked/pre-divisional (S-phase) cell fractions showed MopJ to be absent from the former and present in the latter (Fig. 3b and Supplementary Fig. 5c). The MopJ expression pattern matches that of the TipF flagellar regulator, an unstable protein that is expressed from a GcrA-activated promoter in early S-phase and is subsequently proteolysed in a manner that depends on the ClpXP protease^48^. Measuring LacZ activity in GcrA-depleted cells harbouring the P_*mopJ*_-*lacZ* reporter, we observed a strong reduction after 6 h of depletion relative to *WT* (Fig. 3c) or to GcrA-replete cells (Supplementary Fig. 3b), indicating that GcrA also acts positively on this promoter. In support of this, previous chromatin immunoprecipitation coupled to deep-sequencing experiments revealed that GcrA binds P_*mopJ*_ (Fig. 3d) efficiently *in vivo* and that it harbours N6-methyladenosine (m6A, Fig. 3e) marks, a hallmark of a class of promoters that fire in S-phase and that require methylation for efficient activation and binding of GcrA *in vitro* and *in vivo*^49^,^50^.

Interestingly, MopJ features two terminal alanine residues that resemble (class I) C-terminal recognition motifs of the ClpXP protease^51^ and MopJ was previously found associated with the ClpP protease in pull-down assays^52^. We therefore investigated if ClpXP regulates MopJ abundance *in vivo*. To this end, we used immunoblotting to monitor the abundance of MopJ in cells from which the ClpX ATPase component of the ClpXP proteolytic machine had been depleted (Fig. 3f) or in cells intoxicated with a dominant negative version of ClpX (Fig. 3g)^53^. We found steady-state levels of MopJ to be elevated under these conditions. Moreover, the abundance of MopJ and CtrA, a known ClpXP substrate^18^, is elevated upon disruption of the *clpX* or the *clpP* gene in Δ*socB* mutant cells (Fig. 3h). (Note that inactivation of *clpX* or *clpP* is lethal in *WT* cells, but no longer in Δ*socB* cells, in which the gene encoding the SocB toxin has been deleted^54^). Finally, the levels of a MopJ variant harbouring GFP fused to the C-terminus (MopJ-GFP) expressed from P_*mopJ*_ no longer fluctuate during the cell cycle (Fig. 3I and Supplementary Fig. 5d) and confers motility to a comparable level as the untagged (*WT*) version (Supplementary Fig. 2b).

In sum, a combination of GcrA-controlled synthesis and ClpXP-dependent proteolysis restricts MopJ accumulation to S-phase and ensures that re-synthesis of CtrA and its reinforcement factor MopJ (see above) is linked, thus optimally reinforcing CtrA accumulation and function. Moreover, as MopJ is also induced in stationary phase by (p)ppGpp, an adaptive (nutritional) feed exists into the cell cycle.

### Bipolar localization of MopJ requires the DivJ kinase

**Figure 4:**
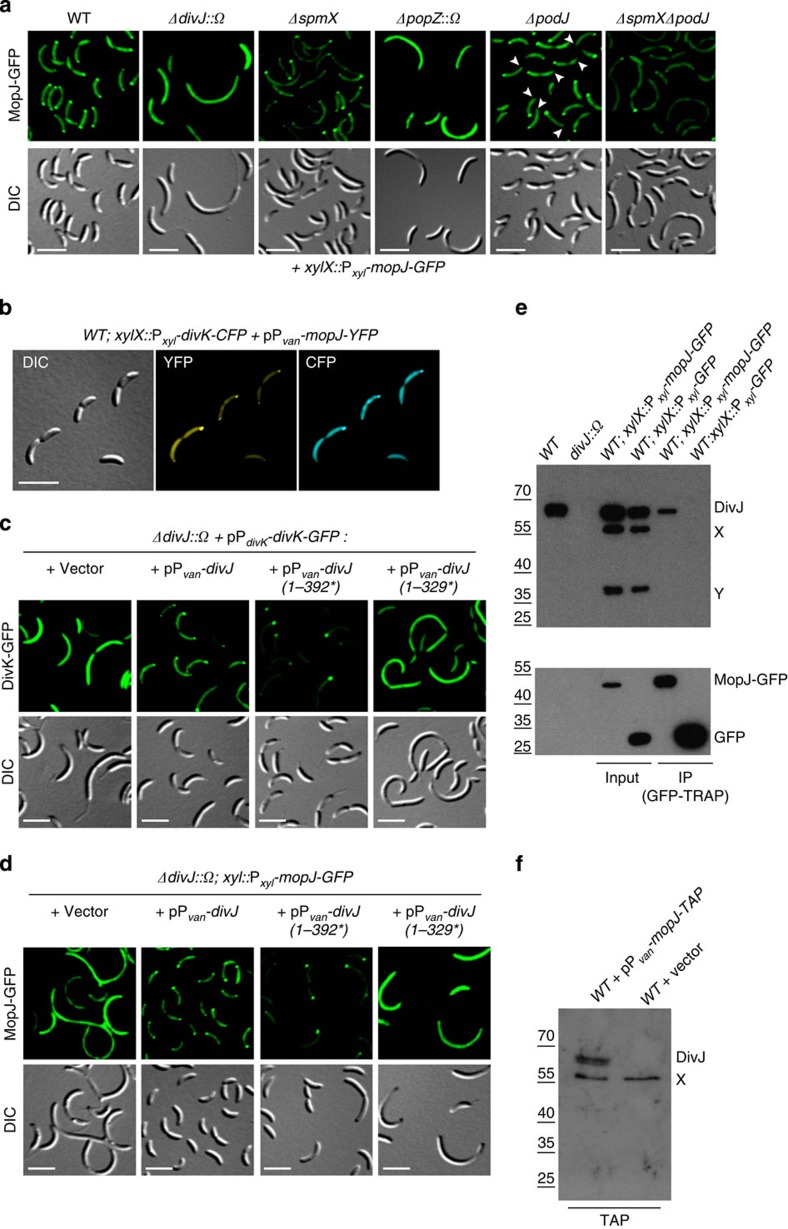
Polar localization of MopJ requires the DivJ kinase and the PopZ polar scaffold. (**a**) Fluorescence and DIC images of live *C. crescentus WT*, Δ*divJ*::*Ω*, Δ*spmX*, Δ*popZ*::Ω, Δ*podJ* or Δ*podJ*Δ*spmX* cells expressing MopJ-GFP under the control of xylose promoter (P_*xyl*_) at the *xylX* locus. White arrowheads point to the flagellated pole. (**b**) Co-localization of DivK-CFP expressed from P_*xyl*_ at the *xylX* locus in *WT* cells and MopJ-YFP expressed from P_*van*_ on pMT374. The corresponding DIC image is also shown. (**c**) Fluorescence and DIC images of Δ*divJ*::Ω cells expressing DivK-GFP fusion from pMR10-divK-GFP and various versions of DivJ, including full-length DivJ, DivJ-392 (short version of *divJ* encoding a truncated protein without histidine kinase domain) or DivJ-329 (short version of *divJ* encoding a truncated protein without kinase and dimerization domains) under the control of the vanillate promoter (P_*van*_) from pMT335. The control harbouring the empty vector is also shown. (**d**) Fluorescence and DIC images of Δ*divJ*::Ω cells expressing MopJ-GFP under the control of P_xyl_ and the different forms of DivJ from pMT335 as described in **c**. (**e**) Co-immunoprecipitation (IP) of DivJ with MopJ-GFP from a GFP-TRAP affinity matrix (ChromoTek GmbH). Precipitated samples were probed for the presence of DivJ and GFP by immunoblotting using antibodies to DivJ (upper) and GFP (lower). Cell lysates used as input are also shown. (**f**) Tandem-affinity-purification (TAP) of MopJ-TAP from P_*van*_ on pMT335 followed by immunoblotting of the samples for the presence of DivJ using polyclonal antibodies to DivJ. In **e** and **f**, X and Y denote nonspecific proteins that react with the antiserum or degradation products.

Superimposed on the temporal regulation of MopJ is its cell cycle-controlled polar sequestration revealed in time-lapse fluorescence microscopic imaging of live *mopJ::mopJ-GFP* cells (Fig. 3j). Although diffuse fluorescence was observed in G1-phase (swarmer) cells, MopJ-GFP localizes first to the stalked pole during the transition into S-phase (stalked) cells and adopted a bipolar localization until MopJ-GFP dispersed from the opposite pole at the time of cell division (Fig. 3j). MopJ-GFP localized in a similar way when expressed constitutively from the xylose-inducible P_*xyl*_ promoter (Fig. 3k), compared with expression from its native promoter in S-phase, providing further evidence that the cell cycle-regulated localization occurs even when MopJ is expressed ectopically. Imaging of MopJ-GFP (Fig. 4a) or GFP-MopJ (Supplementary Fig. 6) expressed from the *xylX* locus in unsynchronized *C. crescentus* cultures also revealed monopolar and bipolar localization patterns. Consistent with the time-lapse imaging, MopJ-GFP is bipolar in pre-divisional cells, but is dispersed from the pole opposite the stalk in deeply constricted cells (Figs 3j,k and 4a), a characteristic also observed for the DivK RR that is phosphorylated by the DivJ kinase (Fig. 1a). Further reinforcing the parallels between MopJ and DivK (bi)polarity, we observed that MopJ-YFP and DivK-CFP co-localize in *C. crescentus WT* cells (Fig. 4b). This prompted us to explore if MopJ and DivK rely on a common localization mechanism. Indeed, in the absence of DivJ, DivK-GFP delocalized^34^ (Fig. 4c) and MopJ-GFP as well (Fig. 4a,d and Supplementary Fig. 7a,b). Moreover, a truncated version (residues 1–392) of DivJ containing the HisKA domain, but lacking the ATPase (HATPase_c) domain, is sufficient to direct MopJ-GFP and DivK-GFP to the stalked pole, but no longer to the opposite pole (Fig. 4c,d and Supplementary Fig. 7a,b). Thus, as was reported for DivK-GFP previously, MopJ-GFP requires DivJ kinase activity to be sequestered to the pole opposite the stalk, but its sequestration to the stalked pole occurs independently of kinase activity^17^ (Fig. 4c,d). A further truncated version of DivJ (residues 1–329) no longer supported polar localization of MopJ-GFP and DivK-GFP (Fig. 4c,d and Supplementary Fig. 7a,b), indicating that the dimerization/phosphor-acceptor (HisKA) domain and/or a determinant encoded from residues 329 to 392 is critical to recruit MopJ and DivK to the stalked pole. By contrast, expression of full-length DivJ from a comparable plasmid reinstated bipolarity upon MopJ-GFP and DivK-GFP (Fig. 4c,d and Supplementary Fig. 7a,b). As both derivatives of DivJ can still localize to the stalked pole (albeit 50% less efficient for the shorter version, Supplementary Fig. 8), this experiment confirms that residues 329–392 of DivJ encode for the localization determinants of MopJ and DivK. We conclude that DivJ integrates the bipolar localization of two dissimilar effectors, MopJ and DivK, to the stalked pole and, in early pre-divisional cells, also to the opposite pole.

**Figure 5:**
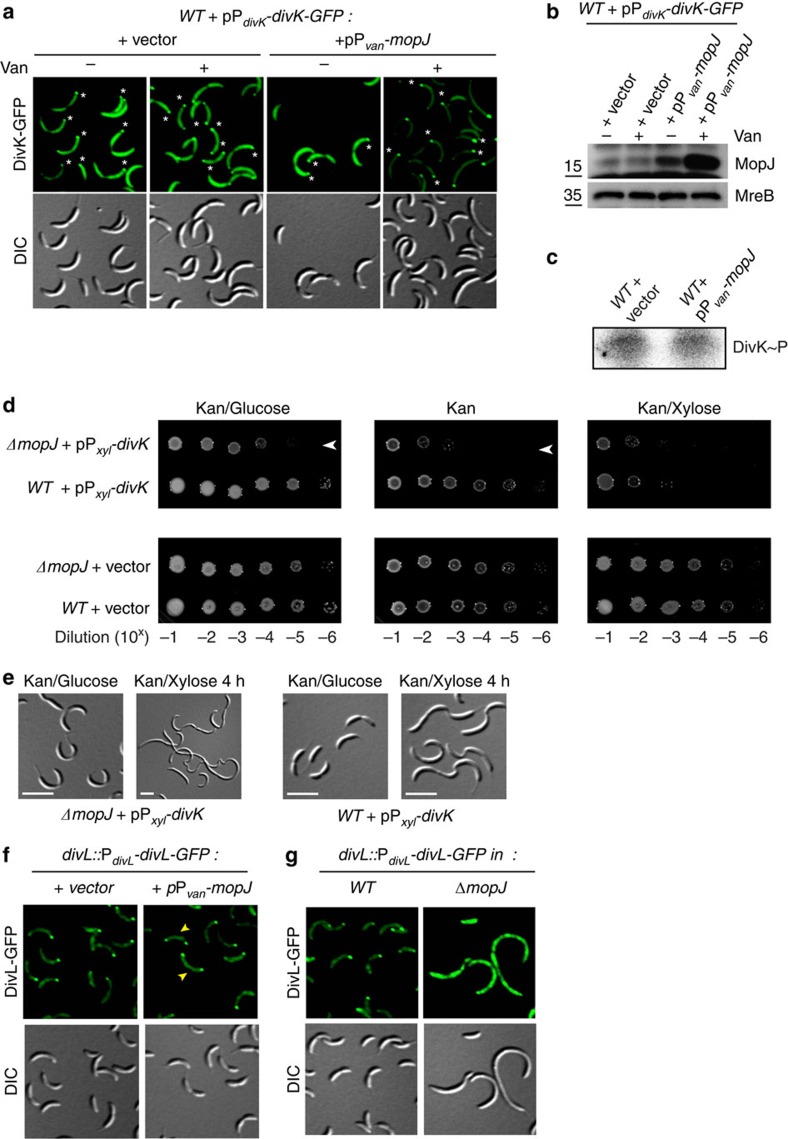
MopJ affects DivK localization and function. (**a**) Fluorescence and DIC imaging of *WT* cells harbouring pMR10-*divK-GFP* and pMT335-P_*van*_*-mopJ* with (+) or without (−) induction (50 μM of vanillate) of MopJ from P_*van*_ on pMT335. The controls harbouring pMR10-*divK-GFP* and the empty vector (pMT335) are also shown. The position of stalks are indicated by a white asterisk. (**b**) Immunoblot showing the steady-state levels of MopJ in strains used in **a**. Steady-state levels of MreB are shown as a loading control. (**c**) *In vivo* phosphorylation of cultures with ^32^P followed by immunoprecipitation reveals DivK∼P levels in *WT* cells harbouring pMT335 or pMT335-*mopJ* (P_*van*_-*mopJ*) after with vanillate (50 μM). (**d**) Effect of Δ*mopJ* mutation on viability in the presence of extra DivK. Efficiency of plating assays of *WT* and Δ*mopJ* cells harbouring the empty vector (pMR10) or the P_*xyl*_-*divK* expression plasmid (pMR10-P_*xyl*_-*divK*). Serial tenfold dilutions of cells plated on PYE agar with kanamycin and glucose (kan+gluc, glucose represses P_*xyl*_), kanamycin (kan, background expression from P_*xyl*_) and kanamycin and xylose (kan+xyl, xylose induces P_*xyl*_) agar. (**e**) DIC (differential interference contrast) imaging of cells described in **d** in glucose or 4 h after induction with xylose (0.3%). The medium was supplemented with kanamycin (5 μg ml^−1^). (**f**) Effect of MopJ overexpression on DivL-GFP localization. The empty vector (pMT335) or the P_*van*_-*mopJ* plasmid (pMT335-*mopJ)* was transformed into *divL*::P_*divL*_-*divL*-*GFP* cells and the resulting cells imaged by DIC and fluorescence microscopy 4 h after induction with vanillate (50 μM). Yellow arrowheads point to cells with bipolar DivL-GFP. (**g**) Effect of *mopJ* deletion on DivL-GFP localization. The *divL*::P_*divL*_-*divL*-*GFP* allele was transduced in *WT* and Δ*mopJ* cells and the resulting cells imaged by DIC and fluorescence microscopy.

In support of the notion that a physical interaction between MopJ and DivJ underlies the localization of MopJ to the stalked pole, co-immunoprecipitation experiments revealed that DivJ is pulled-down with MopJ-GFP from cellular lysates of a *WT* strain expressing MopJ-GFP from P_*xyl*_ (Fig. 4e). By contrast, DivJ was not detectable in comparable pull-downs from lysates of the *WT* strain expressing GFP from P_*xyl*_ (Fig. 4e). Furthermore, DivJ was also recovered with MopJ in tandem-affinity purification experiments^55^ conducted with lysates of *WT* cells expressing MopJ-TAP from P_*van*_ (Fig. 4f). Having shown that MopJ resides in a complex with DivJ, we then tested whether overexpression of MopJ can affect the DivJ-regulated subcellular distribution of the other DivJ-interacting effector DivK^17^,^34^ (Fig. 5a,b). Live-cell imaging of DivK-GFP in cells overexpressing MopJ from P_*van*_ revealed an enhancement of DivK-GFP polar localization and a concomitant reduction of the cytoplasmic fluorescence that correlated with the strong induction of MopJ upon the addition of vanillate (Fig. 5a,b), without affecting DivK∼P levels noticeably by *in vivo* phosphorylation analysis (Fig. 5c). However, we found that Δ*mopJ* cells are sensitized towards elevated DivK levels from a plasmid expressing DivK under the control of P_*xyl*_, suggesting that extra DivK^17^ further curbs the CtrA pathway in Δ*mopJ* cells (in which activation of the CtrA regulon is already disturbed, see above) to inhibit growth (Fig. 5d) and division (Fig. 5e) more than in *WT* cells. Taken together, these experiments show that MopJ binds DivJ and depends on it for its own polar localization, while promoting the polar sequestration of DivK and attenuating its activity.

Examining possible effects on downstream components, we found that MopJ overexpression not only alters the subcellular distribution of DivK, but also that of its interaction partner, the tyrosine kinase DivL^24^ that localizes primarily to the pole opposite the stalk^15^,^22^,^56^ (Fig. 1a). Overexpression of MopJ caused a near fivefold increase in the fraction of bipolar DivL-GFP (expressed from the native promoter at the *divL* locus in lieu of endogenous DivL; Fig. 5f and Supplementary Fig. 9). Conversely, in cells lacking MopJ, DivL-GFP was largely delocalized (Fig. 5g) and a *divL*::Tn*5* mutation (in which Tn5 is inserted upstream of the region encoding the HisKA domain) did not impact MopJ-GFP localization (Supplementary Fig. 10). The previous observation that DivL is delocalized in the absence of DivJ^56^ is consistent with our findings, as we showed above that DivJ is also required for the polarization of MopJ-GFP. Intriguingly, DivL localization is thought to be dependent on the onset of DNA replication^57^. The result that MopJ is required for DivL localization and MopJ expression requires GcrA is noteworthy, as it could provide an explanation for the finding that DivL polarization (and the concomitant re-accumulation of CtrA that this event regulates) requires S-phase entry^57^. GcrA expression and S-phase entry are known to be dependent on the replication initiator DnaA^58^.

In sum, MopJ is both target (via DivJ) and effector (via DivK and DivL) of the *Caulobacter* spatiotemporal cell cycle network and is thus perfectly positioned to reinforce CtrA with the synthesis of both proteins in S-phase and is genetically linked as expression of both proteins is directly induced by GcrA.

### A second localization pathway controls polarization of MopJ

Although DivJ is necessary for the bipolar localization of MopJ, two findings indicate that it is not sufficient for bipolarity. First, MopJ is localized to the pole opposite the stalk (Fig. 4a), where no focus of DivJ is observed^32^. Second, MopJ is still polarized when DivJ is delocalized by inactivation of its localization factor SpmX (that is, in Δ*spmX* mutant cells, Fig. 4a and Supplementary Fig. 11)^3^. Taken together, this indicates that other factors must promote polarization of MopJ. As it is also conceivable that DivJ simply targets MopJ to the membrane or fastens it there, we also explored whether the bipolar scaffolding protein PopZ that forms adhesive patches at both cell poles^26^,^36^,^38^,^39^,^40^ might keep MopJ-GFP polarized. Live-cell imaging revealed that MopJ-GFP is dispersed in Δ*popZ*::Ω mutant cells (Fig. 4a). As PopZ has also been likened to a molecular plug that prevents diffusion away from the cell pole, we predicted that an additional factor promotes MopJ-GFP localization to the pole opposite the stalk. The PodJ polarity protein is known to attract regulatory proteins of CtrA to the pole opposite the stalk^36^,^37^,^59^,^60^. We observed MopJ-GFP to be monopolar in Δ*podJ* mutant cells, unable to assemble into foci at the pole opposite the stalk regardless of whether SpmX was present or not (Fig. 4a and Supplementary Fig. 11). This dependence of MopJ-GFP on PodJ for localization to the flagellated pole not only supports the existence of distinct recruitment mechanisms of MopJ-GFP for each cell pole in *WT* cells, but also provides an explanation of why DivL is no longer localized to the flagellated pole in Δ*podJ* cells^59^. It also points to a possible explanation of how DivJ might influence MopJ localization to the pole opposite the stalk. As the expression of the gene encoding the PerP periplasmic protease that cleaves PodJ is strongly and directly upregulated by CtrA in Δ*divJ*::Ω mutant cells^9^,^10^,^61^, we hypothesize that DivJ promotes the localization of MopJ at the pole opposite the stalk indirectly through PodJ (or another DivJ-dependent protein).

## Discussion

**Figure 6:**
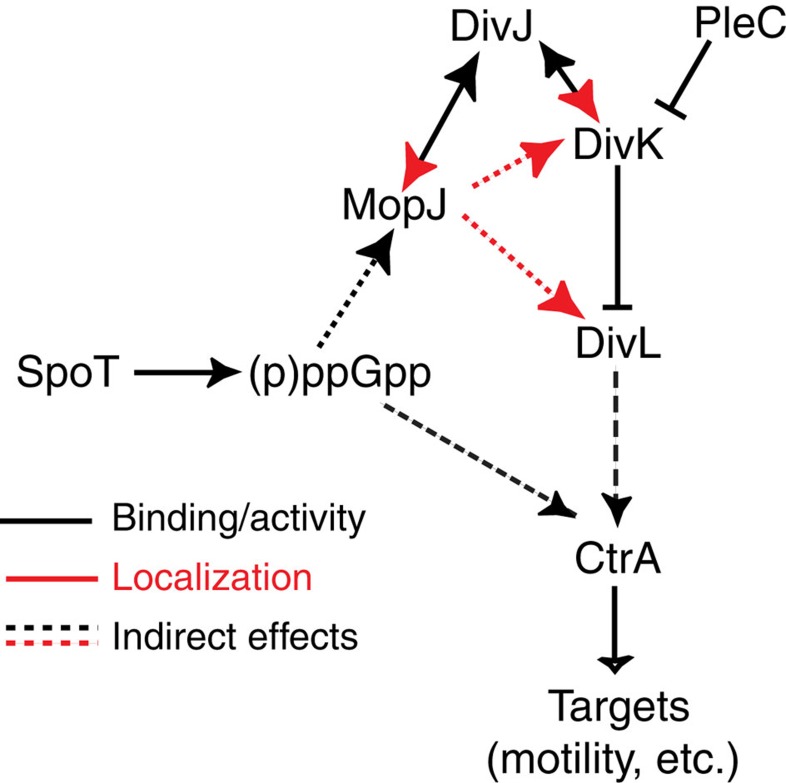
Proposed action of MopJ on the *Caulobacter* cell circuitry. Summary scheme depicting the interactions of MopJ with the cell circuitry as determined in this work. Effects on protein localization are shown in red, whereas interactions shown in black designate direct interactions that generally result in activation (arrow, DivJ-DivK) or inhibition (T-bar, PleC-DivK, DivK-DivL). The scheme also shows the effects of the alarmone (p)ppGpp in inducing MopJ and the maintenance of CtrA^25^,^26^,^28^,^47^ (see Discussion).

A single-domain PAS protein, MopJ, is not only regulated by cell cycle cues, but also integrates nutritional and growth phase regulatory signals to exert topological control over conserved components of a bacterial cell cycle network (Fig. 6). The identification of MopJ enabled us to illuminate two important and hitherto enigmatic cell cycle events. The first conundrum was how the master regulator CtrA can re-accumulate in S-phase^4^, a time when DivK is still phosphorylated and signals the removal of CtrA^30^,^34^. Unless the proteolytic cascade is attenuated and/or the rate of synthesis is potentiated such that it exceeds proteolysis, CtrA accumulation should be suppressed or curbed. We found that the PAS-domain protein MopJ indeed enhances CtrA accumulation and acts on DivK and its target DivL^15^. MopJ re-accumulation is temporally linked with that of CtrA through the S-phase-specific transcriptional activator GcrA that binds the *ctrAP1* and the *mopJ* promoter^8^,^49^,^50^, thus reinforcing CtrA re-synthesis with the coordinated synthesis of its enhancing factor MopJ in S-phase.

By discovering that MopJ acts at the same subcellular site as the CtrA His-Asp phosphorelay components DivJ, DivK and DivL, we were able to identify MopJ as an important missing link connecting DivL polarization to DivJ (Fig. 6) and likely to the onset of DNA replication. MopJ polarization requires the polar organizer PopZ and the DivJ kinase. With the previous findings that polar localization of DivJ is itself dependent on PopZ assembly at polar sites^38^,^40^, our work extends this spatiotemporal regulatory network by showing that MopJ associates with DivJ (Fig. 4), enhances the sequestration of DivK to the cell poles (attenuating DivK function, Fig. 5), governs subcellular localization of DivL and facilitates CtrA accumulation and expression of its target promoters (Fig. 2).

The stationary phase and (p)ppGpp-based induction of MopJ unveils a concerted feed-forward mechanism underlying signal integration during bacterial cell cycle progression, acting on an essential master transcriptional regulator in different phases of growth (Fig. 6). Why MopJ abundance increases in stationary phase remains to be determined, but our experiments suggest that this inductions occurs at the transcriptional level as the activity of the *mopJ* promoter is elevated in stationary phase and is inducible in nutrient replete conditions when (p)ppGpp levels are raised artificially in exponential phase (Fig. 2). As (p)ppGpp signals nutrient starvation by globally reprogramming RNA polymerase and is induced in stationary phase in many bacteria^46^, the induction of MopJ is likely mediated at the level of promoter activity. It is also possible that MopJ synthesis is further reinforced at the level of protein stability by inhibiting ClpXP-mediated proteolysis (Fig. 3). It is not obvious why MopJ should be induced in stationary phase when nutrients are exhausted to enhance the cell cycle regulator CtrA. However, it is known that CtrA levels are maintained in the presence of (p)ppGpp^26^,^28^,^47^, and our results raise the possibility that this effect is (at least partially) mediated by (induced) MopJ. In light of the fact that the number of cells undergoing DNA replication is strongly diminished in stationary phase, with cells accumulating predominantly in G1 and G2 phase (Fig. 1), along with the fact that CtrA acts on replication control^4^,^12^, we suspect that CtrA is responsible or at least contributes to this replication arrest. In support of this, cells no longer exhibit a normal stationary phase arrest in the absence of MopJ (Fig. 1), and the abundance of CtrA is strongly reduced under these conditions (Fig. 2). Such stationary-phase-induced replication arrest may be further accentuated by other (p)ppGpp-dependent mechanisms, such as the reduction of the replication initiator DnaA, an unstable protein^62^,^63^, when (p)ppGpp levels are high^26^,^28^,^47^. It is also noteworthy that in contrast to this specific cell cycle control mechanism on the level of a master regulator, (p)ppGpp induces a general growth arrest and a state of antibiotic tolerance (known as persistence) in *E. coli* and likely other bacteria through a shutdown of macromolecular synthesis by type II toxin–antitoxin systems that are activated indirectly upon accumulation of (p)ppGpp^64^.

(p)ppGpp-induced replication arrest mechanism(s) are also operational in other α-Proteobacteria^25^, as a nutritional down-shift induces a cell cycle arrest in *S. meliloti*^65^. Although CtrA is not known to bind the origin of replication in *Sinorhizobium fredii* directly^10^, CtrA may inhibit replication initiation indirectly as was recently also suggested to be the case for *Caulobacter*^13^ and reinforcement of CtrA by induced MopJ presents an appealing and conceivable possibility. This is mechanistically distinct from the (p)ppGpp-based replication arrest at the level of DNA primase described for the Gram-positive soil bacterium *Bacillus subtilis*^66^, a member of the Firmicutes, clearly showing that evolution has found elegant solutions to the same problem.

## Methods

### Growth conditions

*Caulobacter crescentus* NA1000 (ref. 43) and derivatives were cultivated at 30 °C in peptone yeast extract (PYE)-rich medium or in M2 minimal salts plus 0.2% glucose (M2G) supplemented by 0.4% liquid PYE^5^. *E. coli* S17-1 and EC100D (Epicentre Technologies) were cultivated at 37 °C in Luria Broth (LB)-rich medium. 1.5% agar was added into M2G or PYE plates and motility was assayed on PYE plates containing 0.3% agar. Antibiotic concentrations used for *C. crescentus* include kanamycin (solid: 20 μg ml^−1^; liquid: 5 μg ml^−1^), tetracycline (1 μg ml^−1^), spectinomycin (liquid: 25 μg ml^−1^), spectinomycin/streptomycin (solid: 30 and 5 μg ml^−1^, respectively), gentamycin (1 μg ml^−1^) and nalidixic acid (20 μg ml^−1^). When needed, D-xylose or sucrose was added at 0.3% final concentration, glucose at 0.2% final concentration and vanillate 500 or 50 μM final concentration. For the experiments in stationary phase in PYE, cultures with an OD_660nm_ >1.4 were used.

Swarmer cell isolation, electroporation, biparental mating and bacteriophage φCr30-mediated generalized transduction were performed as described in ref. 5. Briefly, swarmer cells were isolated by Ludox or Percoll density-gradient centrifugation at 4 °C, followed by three washes and final re-suspension in pre-warmed (30 °C) M2G. Electroporation was done using 1 mm gap electroporation cuvettes at 1.5 kV in an Eppendorf 2510 electroporator and 6 ml exponential phase cells that had been washed three times in sterile water. Biparental matings were done using exponential phase *E. coli* S17-1 donor cells and *Caulobacter* recipient cells washed in PYE and mixed at 1:10 ratio on a PYE plate. After 4–5 h of incubation at 30 °C, the mixture of cells was plated on PYE harbouring nalidixic acid (to counter select *E. coli*) and the antibiotic that the conjugated plasmid confers resistance to. Generalized transductions were done by mixing 50 μl ultraviolet-inactivated transducing lysate with 500 μl exponential phase recipient cells, incubation for 2 h, followed by plating on PYE containing antibiotic to elect for the transduced DNA.

### Bacterial strains, plasmids and oligonucleotides

Bacterial strains, plasmids and oligonucleotides used in this study are listed and described in Supplementary Tables 3–5, respectively.

### Co-immunoprecipitation

When the culture (50 ml) reached OD_660nm_=0.4–0.6 in the presence of xylose, cells were harvested by centrifugation at 6,000*g* for 10 min. The pellet was then washed in 10 ml of dilution/wash buffer (10 mM Tris-HCl (pH 7.5); 150 mM NaCl; 0.5 mM EDTA) and lysed for 15 min at room temperature in 1 ml of lysis buffer (dilution/wash buffer+0.5% NP40, 10 mM MgCl_2_, two protease inhibitor tablets (for 50 ml of buffer; Complete EDTA-free, Roche), 1 × Ready-Lyse lysozyme (Epicentre), 50 U of DNase I )Roche)). Cellular debris was removed by centrifugation at 7,000*g* for 15 min at 4 °C. The supernatant was incubated for 1 h (or overnight) at 4 °C with GFP-Trap_A beads (ChromoTek GmbH) previously washed three times with 500 μl of dilution/wash buffer. The sample was then centrifuged at 2,500*g* for 2 min at 4 °C and the supernatant was removed. The beads were washed three times with 500 μl of dilution/wash buffer and finally resuspended in 2 × SDS sample buffer (50 mM Tris–HCl (pH 6.8), 2% SDS, 10% glycerol, 1% β-mercaptoethanol, 12.5 mM EDTA, 0.02% Bromophenol Blue), heated to 95 °C for 10 min and stored at −20 °C.

### Tandem affinity purification (TAP)

The TAP procedure was based on that described by ref. 55. Briefly, when the culture (1 l) reached OD_660nm_=0.4–0.6 in the presence of 50 μM vanillate, cells were harvested by centrifugation at 6,000*g* for 10 min. The pellet was then washed in 50 ml of buffer I (50 mM sodium phosphate at pH 7.4, 50 mM NaCl, 1 mM EDTA) and lysed for 15 min at room temperature in 10 ml of buffer II (buffer I+0.5% n-dodecyl-β-D-maltoside, 10 mM MgCl_2_, two protease inhibitor tablets (for 50 ml of buffer II; Complete EDTA-free, Roche), 1 × Ready-Lyse lysozyme (Epicentre), 500 U of DNase I (Roche)). Cellular debris was removed by centrifugation at 7,000*g* for 20 min at 4 °C. The supernatant was incubated for 2 h at 4 °C with IgG Sepharose beads (GE Healthcare Biosciences) that had been washed once with IPP150 buffer (10 mM Tris-HCl at pH 8, 150 mM NaCl, 0.1% NP40). After incubation, the beads were washed at 4 °C three times with 10 ml of IPP150 buffer and once with 10 ml of TEV cleavage buffer (10 mM Tris-HCl at pH 8, 150 mM NaCl, 0.1% NP40, 0.5 mM EDTA, 1 mM DTT). The beads were then incubated overnight at 4 °C with 1 ml of TEV solution (TEV cleavage buffer with 100 U of TEV protease per millilitre (Promega)) to release the tagged complex. CaCl_2_ (3 μM) was then added to the solution. The sample with 3 ml of calmodulin-binding buffer (10 mM β-mercaptoethanol, 10 mM Tris-HCl at pH 8, 150 mM NaCl, 1 mM magnesium acetate, 1 mM imidazole, 2 mM CaCl_2_, 0.1% NP40) was incubated for 1 h at 4 °C with calmodulin beads (GE Healthcare Biosciences) that previously had been washed once with calmodulin-binding buffer. After incubation, the beads were washed three times with 10 ml of calmodulin-binding buffer and eluted five times with 200 μl IPP150 calmodulin elution buffer (calmodulin-binding buffer substituted with 2 mM EGTA instead of CaCl_2_). The eluates were then concentrated using Amicon Ultra-4 spin columns (Ambion).

### β-Galactosidase assays

β-Galactosidase assays were performed at 30 °C as described previously^3^. 50 μl of cells grown in PYE at OD_660nm_=0.1–0.6 were lysed with chloroform and mixed with 750 μl of Z buffer (60 mM Na_2_HPO_4_, 40 mM NaH_2_PO_4_, 10 mM KCl and 1 mM MgSO_4_ heptahydrate). 200 μl of ONPG (4 mg ml^−1^ *o*-nitrophenyl-β-D-galactopyranoside in 0.1 M KPO_4_ pH 7.0) was added and the reaction timed. When a medium-yellow colour developed the reaction was stopped with 400 μl of 1 M Na_2_CO_3_. The OD_420nm_ of the supernatant was determined and the units were calculated with the equation: *U*=(OD_420nm_ × 1000)/(OD_660nm_ × time (in min) × volume of culture (in ml)). For the stationary phase experiments, stationary phase cultures were diluted in PYE to obtain an OD_660nm_=0.1–0.6 and quickly lysed with chloroform. Following steps were performed as above. For GcrA depletion experiments in PYE using strain NA1000 Δ*gcrA*::Ω *xylX*::P*xyl*-*gcrA*, PYE supplemented with 0.3% xylose overnight cultures were harvested and washed three times with PYE, and then restarted in appropriate PYE supplemented with 0.3% xylose or PYE supplemented with 0.2% glucose medium for 0, 1.5, 3, 4.5 and 6 h at 30 °C. Experimental values represent the averages of three independent experiments.

### MopJ purification and production of antibodies

MopJ-short protein, lacking the first N-terminal 45 residues, was expressed from pET28a in *E. coli* Rosetta (DE3)/pLysS (Novagen) and purified under native conditions using Ni^2+^ chelate chromatography. A 5-ml overnight culture was diluted into 1 l of pre-warmed LB. When cells reached OD_660nm_=0.3–0.4, 1 mM isopropyl-β-D-thiogalactoside was added to the culture and growth continued. After 3 h, cells were pelleted and resuspended in 25 ml of lysis buffer (10 mM Tris HCl (pH 8), 0.1 M NaCl, 1.0 mM b-mercaptoethanol, 5% glycerol, 0.5 mM imidazole Triton X-100 0.02%). Cells were sonicated (Sonifier Cell Disruptor *B-30*; Branson Sonic Power. Co.) on ice using 12 bursts of 20 s at output level 5.5. After centrifugation at 4,300*g* for 20 min, the supernatant was loaded onto a column containing 5 ml of Ni-NTA agarose resin pre-equilibrated with lysis buffer. Column was rinsed with lysis buffer, 400 mM NaCl and 10 mM imidazole, both prepared in lysis buffer. Fractions were collected (in 300 mM Imidazole buffer, prepared in lysis buffer) and used to immunize New Zealand white rabbits (Josman LLC).

### Immunoblot analysis

Pelleted cells were re-suspended in 1 × SDS sample buffer (50 mM Tris-HCl (pH 6.8), 2% SDS, 10% glycerol, 1% β-mercaptoethanol, 12.5 mM EDTA, 0.02% Bromophenol Blue), heated to 95 °C for 10 min and stored at −20 °C. Protein samples were separated by SDS–polyacrylamide gel electrophoresis and blotted on polyvinylidenfluoride membranes (Merck Millipore). Membranes were blocked for 1 h with Tris-buffered saline (TBS), 0.05% Tween-20 and 5% dry milk and then incubated for an additional 1 h with the primary antibodies diluted in TBS, 0.05% Tween-20, 5% dry milk. The different polyclonal antisera to PilA (1:5,000 dilution), FljK (1:10,000), CtrA (1:10,000) and SciP (1:2,000) were used as described before^10^. The other antibodies were used at following dilutions: anti-GcrA (1:10,000)^8^, anti-MreB (1:20,000)^67^, anti-DivJ (1:10,000)^32^ and anti-MopJ (1:4,000). Commercial monoclonal (Roche, CH) and polyclonal antibodies to GFP were used at 1:5,000 and 1:10,000 dilutions, respectively. The membranes were washed four times for 5 min in PBS and incubated for 1 h with the secondary antibody (HRP-conjugated donkey anti-rabbit antibody, Jackson ImmunoResearch) diluted 1:20,000 in TBS, 0.05% Tween-20 and 5% dry milk. The membranes were finally washed again four times for 5 min in PBS and revealed with Immobilon Western Blotting Chemoluminescence HRP substrate (Merck Millipore) and Super RX-film (Fujifilm).

### Microscopy

PYE or M2G cultivated cells in exponential growth phase were immobilized using a thin layer of 1% agarose. For time-lapse experiments, synchronized cells were immobilized using a thin layer of 1% agarose in M2G supplemented with 0.4% PYE. Fluorescence and contrast microscopy images were taken with an Alpha Plan-Apochromatic × 100/1.46 DIC(UV) VIS-IR oil objective on an Axio Imager M2 microscope (Zeiss) with acquisition at 535 nm (enhanced green fluorescent protein), 580 nm (yellow fluorescent protein (YFP)) and 480 nm (cyan fluorescent protein (CFP); Visitron Systems GmbH) and a Photometrics Evolve camera (Photometrics) controlled through Metamorph V7.5 (Universal Imaging). Images were processed using Metamorph V7.5.

### Fluorescence-activated cell sorting

FACS experiments were performed as described previously^50^. Cells in exponential growth phase (OD_660nm_=0.3–0.6) or in stationary phase (diluted to obtain an OD_660nm_=0.3–0.6), cultivated in M2G, were fixed in ice-cold 70% ethanol solution. Fixed cells were re-suspended in FACS staining buffer, pH 7.2 (10 mM Tris-HCl, 1 mM EDTA, 50 mM NaCitrate, 0.01% Triton X-100) and then treated with RNase A (Roche) at 0.1 mg ml^−1^ for 30 min at room temperature. Cells were stained in FACS staining buffer containing 0.5 μM of SYTOX Green nucleic acid stain solution (Invitrogen) and then analysed using a BD Accuri C6 flow cytometer instrument (BD Biosciences). Flow cytometry data were acquired and analysed using the CFlow Plus V1.0.264.15 software (Accuri Cytometers Inc.). 20,000 cells were analysed from each biological sample. The forward scattering (FSC-A) and Green fluorescence (FL1-A) parameters were used to estimate cell sizes and cell chromosome contents, respectively. Experimental values represent the averages of three independent experiments. Relative chromosome number was directly estimated from the FL1-A value of NA1000 cells treated with 20 μg ml^−1^ Rifampicin for 3 h at 30 °C, done in ref. 50. Rifampicin treatment of cells blocks the initiation of chromosomal replication, but allows ongoing rounds of replication to finish.

### *In vivo* phosphorylation-immunoprecipitation experiments

*In vivo* phosphorylation measurements by ^32^P labelling of *WT* cultures harbouring pMT335 or pMT335-*mopJ* after induction with vanillate (50 μM), followed by immunoprecipitation with antibodies to DivK as described in the study by Radhakrishnan *et al*.^3^. Briefly, a freshly grown colony was picked from a PYE plate, washed with M5G medium lacking phosphate and was cultivated overnight in M5G with 0.05 mM phosphate to an optical density of 0.3 at 660 nm. One millilitre of culture was labelled for 4 min at 28 °C using 30 μCi of γ-[32P]ATP. Upon lysis, proteins were immunoprecipitated with 3 μl of anti-DivK antiserum and Protein A agarose (Roche, CH) and the precipitates were resolved by SDS–polyacrylamide gel electrophoresis and radiolabelled DivK was quantified.

### Strain constructions

#### NA1000 Δ*mopJ*

Deletions were introduced using SacB-based counterselection using 3% sucrose. Briefly, pNPTS138-Δ*mopJ*-KO was first introduced into NA1000 (*WT*) by intergeneric conjugation and then plated on PYE harbouring kanamycin (to select for recombinants) and nalidixic acid to counter select *E. coli* donor cells^5^. A single homologous recombination event at the *CCNA_00999* locus of kanamycin-resistant colonies was verified by PCR. The resulting strain was grown to stationary phase in PYE medium lacking kanamycin. Cells were then plated on PYE supplemented with 3% sucrose and incubated at 30 °C. Single colonies were picked and transferred in parallel onto plain PYE plates and PYE plates containing kanamycin. Kanamycin-sensitive cells, which had lost the integrated plasmid due to a second recombination event, leaving a deleted version of *mopJ* behind (Δ*mopJ)*, were then identified for disruption of the *mopJ* locus by PCR.

#### NA1000 Δ*spmX*Δ*podJ*

pNPTS138-Δ*spmX*-KO^3^ was first introduced into NA1000 Δ*podJ*^36^ by intergeneric conjugation and then plated on PYE harbouring kanamycin (to select for recombinants) and nalidixic acid to counter select *E. coli* donor cells. A single homologous recombination event at the *CCNA_02255* locus of kanamycin-resistant colonies was verified by PCR. The resulting strain was treated as above.

#### NA1000 *mopJ::himar1*

*mopJ::himar1* allele (*himar1* insertion at nt 1082252, Kan^R^) was transduced into NA1000 from NS61 using φCr30-mediated generalized transduction.

#### Strains harbouring pMT335 or pMT335-derived plasmids

pMT335 or pMT335-*mopJ* was introduced by electroporation and then plated on PYE plates harbouring gentamycin.

#### NA1000 Δ*socB* Δ*clpP*::Ω and Δ*socB* Δ*clpX*::Ω

The Δ*clpP*::Ω (Spc^R^) or Δ*clpX*::Ω (Spc^R^)^19^ allele was introduced into NA1000 Δ*socB* as described in ref. 54 by φCr30-mediated transduction followed by selection on plates harbouring spectinomycin.

#### NA1000 *xylX::*P_*xyl*_ -clpX*

pMO88 (ref. 68) was introduced into NA1000 by electroporation and then plated on PYE harbouring tetracycline. The resulting strain was grown to stationary phase in PYE medium containing tetracycline.

#### NA1000 *ctrA*::*ctrA-M2* and Δ*mopJ ctrA*::*ctrA-M2*

The *ctrA*::*ctrA-M2* allele^45^ was introduced into NA1000 or Δ*mopJ* by φCr30-mediated transduction on PYE plates harbouring kanamycin.

#### NA1000 Δ*cpdR*::*tetR* and Δ*mopJ* Δ*cpdR*::*tetR*

The Δ*cpdR*::*tet* (Tet^R^) allele was transduced from Δ*cpdR*:: *tet*^16^ into NA1000 Δ*mopJ* followed by selection on PYE plates harbouring tetracycline.

#### Strains harbouring pMR10 or derivatives

Plasmids were introduced by electroporation, followed by selection of transformants on PYE plates harbouring tetracycline (pMR20) or kanamycin (pMR10).

#### Strains harbouring *xylX*::P_*xyl*_ *-mopJ-GFP*

pCWR282 (pXGFP4-*mopJ*) was introduced into *C. crescentus* by electroporation and then plated on PYE harbouring kanamycin (to select for recombinants). A single homologous recombination event at the *xyl* locus of kanamycin-resistant colonies was verified by PCR.

#### Strains harbouring *xylX*::P_*xyl*_ *-divJ-GFP*, *xylX*::P_*xyl*_ *-divJ392-GFP* or *xylX*::P_*xyl*_ *-divJ329-GFP*

pXGFP4-*divJ*, pXGFP4-*divJ392* or pXGFP4-*divJ329* were introduced into *C. crescentus* by electroporation and then plated on PYE harbouring kanamycin (to select for recombinants). A single homologous recombination event at the *xyl* locus of kanamycin-resistant colonies was verified by PCR.

#### Strains harbouring pMR10-*divK-GFP* and pMT335 or pMT335-*mopJ*

pMT335-*mopJ* was introduced into strains harbouring pMR10-*divK-GFP* (P_*divK*_*-divK-GFP*) by electroporation and then plated on PYE harbouring kanamycin and gentamycin.

#### NA1000 *mopJ*::P_*mopJ*_ *-mopJ-GFP*

pGFP4-*mopJ* was introduced into NA1000 Δ*mopJ* by electroporation and then plated on PYE harbouring kanamycin (to select for recombinants). A single homologous recombination event at the *mopJ* locus of kanamycin-resistant colonies was verified by PCR.

#### NA1000 *xylX*::P_*xyl*_ *-GFP-mopJ*

pXGFP4-C1-*GFP*-*mopJ* was introduced into NA1000 by electroporation and then plated on PYE harbouring kanamycin. A single homologous recombination event at the *xyl* locus of kanamycin-resistant colonies was verified by PCR.

#### NA1000 *xylX*::P_*xyl*_ *-divK-CFP* P_*van*_ *-mopJ-YFP*

pMT374-*mopJ-YFP* was introduced into NA1000 *xylX*::P_*xyl*_*-divK-CFP*^17^ by electroporation and then selected on PYE plates harbouring tetracycline and kanamycin.

#### Strains harbouring p*lacZ*290 or derivatives

p*lacZ*290 or derivatives^10^ were introduced into *C. crescentus* strains by electroporation and then plated on PYE harbouring tetracycline.

#### NA1000 *xylX*::pXTCYC4 p*lacZ*290-P*mopJ*

p*lacZ*290-P*mopJ* was introduced into NA1000 *xylX*::pXTCYC4 (ref. 47) by electroporation and then plated on PYE harbouring gentamycin and tetracycline.

#### NA1000 harbouring *xylX*::pXTCYC4^47^, *xylX*::pXTCYC4-*relA'-FLAG* or *xylX*::pXTCYC4-*relA'(E335Q)-FLAG* and p*lacZ*290-P*mopJ*

p*lacZ*290-P*mopJ* was introduced into NA1000 *xylX*::pXTCYC4 (ref. 47), *xylX*::pXTCYC4-*relA'-FLAG or xylX*::pXTCYC4-*relA'(E335Q)-FLAG*^47^ by electroporation and then plated on PYE harbouring gentamycin and tetracycline.

#### NA1000 *divL*::*divL-GFP* and Δ*mopJ divL*::*divL-GFP*

*divL-GFP::kan* allele^69^ (Kan^R^) was transduced into NA1000 Δ*mopJ* using φCr30-mediated generalized transduction.

#### *divL*::*divL-GFP* strains harbouring pMT335 or derivatives

Plasmids were introduced into *divL*::*divL-GFP* strains by electroporation and then plated on PYE harbouring kanamycin and gentamycin.

#### EC100D pET28a-*mopJshort*

pET28a-*mopJshort* was introduced into EC100D by electroporation and then plated on LB harbouring kanamycin.

### Plasmid constructions

#### pCWR296 (pNPTS138**-Δ*mopJ-KO*)

The plasmid construct used for *mopJ* (*CCNA_00999*) deletion was made by PCR amplification of two fragments. The first, a 568-bp fragment (nt 1081464–1082022, NA 1000 genome coordinates, flanked by an *Eco*RI site at the 5′end and a *Bam*HI at the 3′end) was amplified using primers delmopJ_1-BamHI and delmopJ_1-EcoRI (sequences for all the primers used in this work can be found in Supplementary Table 5). The second, a 447-bp fragment (nt 1082446–1082880, flanked by a *Bam*HI site at the 5′end and a *Hin*dIII site at the 3′end) was amplified using primers delmopJ_2-HindIII and delmopJ_2-BamHI. These two fragments were first digested with appropriate restriction enzymes and then triple ligated into pNTPS138 (M.R.K. Alley, Imperial College London, unpublished) that had been previously restricted with *Hind*III and *Eco*RI. This construct deletes 423 nt of the *mopJ*-coding sequence (nt 1082023–1082445, or codons 14–154 of the annotated CCNA_00999 coding sequence).

#### pMT335-*mopJ* (pUG16)

The *mopJ*-coding sequence (nt 1081990–1082481, NA1000 genome) was PCR amplified from the NA1000 strain using the mopJ-NdeI and mopJ-EcoRI primers. This fragment was digested with *Nde*I/*Eco*RI and cloned into *Nde*I/*Eco*RI-digested pMT335 (ref. 70).

#### pMT335-*divJ*

The *divJ*-coding sequence (nt 1220499–1222292) was PCR amplified from the NA1000 strain using the divJ-NdeI and divJ-MunI primers. This fragment was digested with *Nde*I/*Mun*I and cloned into *Nde*I/*Eco*RI-digested pMT335.

#### pMT335-*divJ392*

The *divJ392*-coding sequence (short *divJ* form without sequence encoding for kinase domain, nt 1220499–1221674) was PCR amplified from the NA1000 strain using the divJ-NdeI and divJ392-MunI primers. This fragment was digested with *Nde*I/*Mun*I and cloned into *Nde*I/*Eco*RI-digested pMT335.

#### pMT335-*divJ329*

The *divJ329*-coding sequence (short *divJ* form without sequence encoding for dimerization and kinase domains, nt 1220499–1221485) was PCR amplified from the NA1000 strain using the divJ-NdeI and divJ329-MunI primers. This fragment was digested with *Nde*I/*Mun*I and cloned into *Nde*I/*Eco*RI-digested pMT335.

#### pXGFP4-*divJ*

The *divJ*-coding sequence without stop codon (nt 1220499–1222289) was PCR amplified from the NA1000 strain using the divJ-NdeI and divJ_fusion-MunI primers. This fragment was digested with *Nde*I/*Mun*I and cloned into *Nde*I/*Eco*RI-digested pXGFP4 (M.R.K. Alley, Imperial College London, unpublished).

#### pXGFP4-*divJ392*

The *divJ392*-coding sequence (short *divJ* form without sequence encoding for kinase domain, nt 1220499–1221674) was PCR amplified from the NA1000 strain using the divJ-NdeI and divJ392fusion-MunI primers. This fragment was digested with *Nde*I/*Mun*I and cloned into *Nde*I/*Eco*RI-digested pXGFP4.

#### pXGFP4-*divJ329*

The *divJ329*-coding sequence (short *divJ* form without sequence encoding for dimerization and kinase domains, nt 1220499–1221485) was PCR amplified from the NA1000 strain using the divJ-NdeI and divJ329fusion-MunI primers. This fragment was digested with *Nde*I/*Mun*I and cloned into *Nde*I/*Eco*RI-digested pXGFP4.

#### pET28a-*mopJshort*

The *mopJ*-coding sequence lacking the first 132 bp from the start codon (nt 1082122–1082481) was PCR amplified from the NA1000 strain using the mopJshort-NdeI and mopJ-EcoRI primers. This fragment was digested with *Nde*I/*Eco*RI and cloned into *Nde*I/*Eco*RI-digested pET28a (Novagen).

#### pCWR282 (pXGFP4-*mopJ*)

The *mopJ*-coding sequence without stop codon (nt 1081990–1082478) was PCR amplified from the NA1000 strain using the mopJ-NdeI and mopJfusion-BamHI primers. This fragment was digested with *Nde*I/*Bam*HI and cloned into *Nde*I/*Bam*HI-digested pXGFP4.

#### pGFP4-*mopJ*

The *mopJ*-coding sequence without stop codon and the upstream region of 640 bp (nt 1081990–1082478) were PCR amplified from the NA1000 strain using the mopJ-NheI and mopJfusion-BamHI primers. This fragment was digested with *Nhe*I/*Bam*HI and cloned into pXGFP4, previously digested with *Nhe*I/*Bam*HI to remove the P*xylX* locus.

#### pXGFP4-C1-*GFP-mopJ*

The *mopJ*-coding sequence (nt 1081993–1082481), in which start codon ATG was replaced with GTC, was PCR amplified from the NA1000 strain using the mopJfusion-BglII and mopJ-EcoRI primers. This fragment was digested with *Bgl*II/*Eco*RI and cloned into *Bgl*II/*Eco*RI-digested pXGFP4-C1 (M.R.K. Alley, Imperial College London, unpublished).

#### p*lacZ*290-P*mopJ*

The *mopJ* promoter region (−228 to +78 relative to the ATG, nt 1081990–1081992) was PCR amplified using PmopJ-EcoRI and PmopJ-XbaI primers using NA1000 chromosomal DNA as a template. This fragment was digested by *Eco*RI*-Xba*I and cloned into *Eco*RI*-Xba*I-digested p*lacZ*290 promoter probe vector.

## Author contributions

S.S., M.B., S.K.R. and P.H.V conceived and designed the experiments. S.S., M.B., L.T., S.K.R. and P.H.V. performed the experiments. S.S., M.B., S.K.R. and P.H.V analysed the data. S.S. and P.H.V. wrote the paper.

## Additional information

**How to cite this article:** Sanselicio, S. *et al*. Topological control of the *Caulobacter* cell cycle circuitry by a polarized single-domain PAS protein. *Nat. Commun.* 6:7005 doi: 10.1038/ncomms8005 (2015).

## Supplementary Material

Supplementary Information Supplementary Figures 1-11, Supplementary Tables 1-5 and Supplementary References
