# Supplementary material for: Topological control of the Caulobacter cell cycle circuitry by a polarized single-domain PAS protein
Source: Nat Commun. 2015 May 8;6:7005. doi: 10.1038/ncomms8005 (PMC4432633; doi:10.1038/ncomms8005)
Supplement: Supplementary Information — Supplementary Figures 1-11, Supplementary Tables 1-5 and Supplementary References [file ncomms8005-s1.pdf]

Supplementary Information

Supplementary Figures

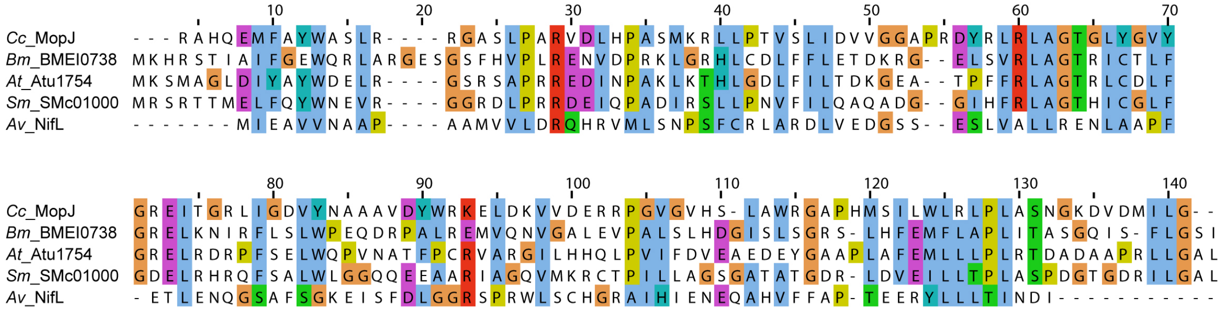

Supplementary Figure 1. Conservation of MopJ. Alignment of MopJ from *Caulobacter crescentus* with that homologous proteins from *Brucella melitensis* (BMEI0738) (reference genome: *Brucella melitensis* bv. 1 str. 16M), *Agrobacterium tumefaciens* (Atu1754) (reference genome: *Agrobacterium fabrum* str. C58) and *Sinorhizobium meliloti* (SMc01000) (reference genome: *Sinorhizobium meliloti* 1021). The alignment of homologous PAS5 domains in MopJs with the more distantly related PASB domain of NifL from *Azotobacter vinelandii* is also shown.

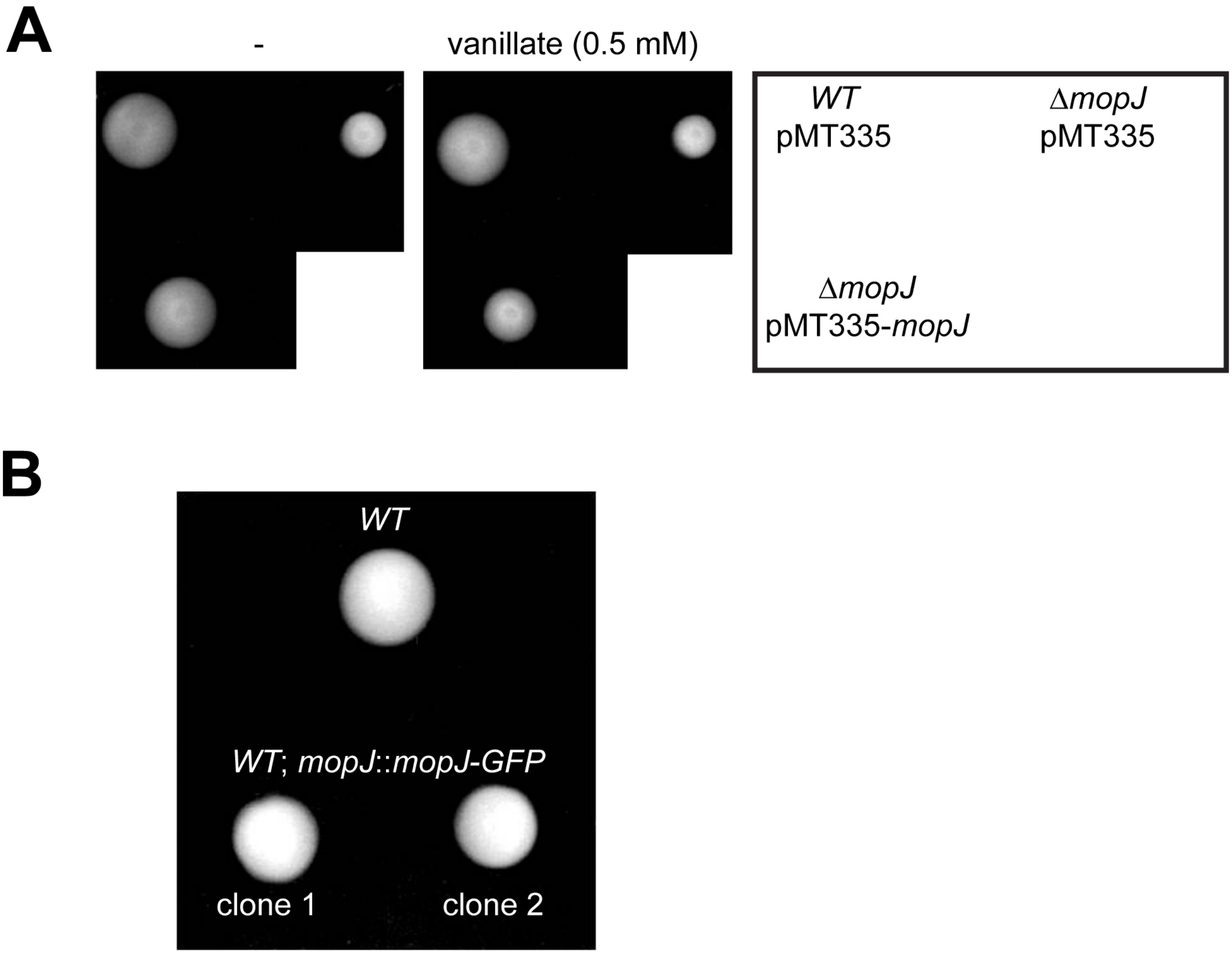

Supplementary Figure 2. Complementation of the Δ*mopJ* motility defect. A. Motility complementation assay of Δ*mopJ* cells on swarm agar plates. Constitutive low expression [without induction (-)] of *mopJ* from P_{van} on pMT335 is sufficient to complement the motility defect of Δ*mopJ* cells. **B.** Cells harboring the *mopJ::mopJ-GFP* allele in place of *WT mopJ* show near *WT* motility. Two clones are shown.

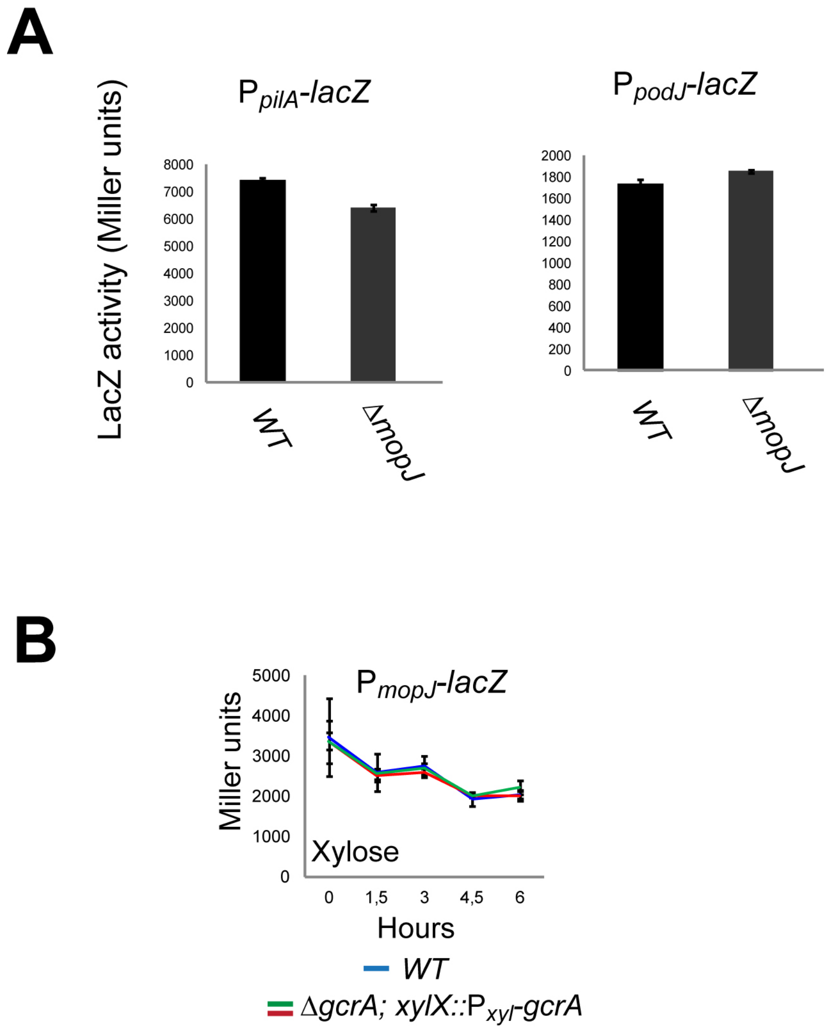

Supplementary Figure 3. Control of transcription by *mopJ* and of *mopJ* by GcrA. A. LacZ measurements of P_{podJ}-*lacZ* (control) and P_{pilA}-*lacZ* complementing the data shown in Figure 2A in the main text. **B.** Positive control of the experiment described in Figure 3C in the main text. Promoter-probe assays of a transcriptional reporter carrying the *mopJ* promoter fused to *lacZ (*P_{mopJ}-*lacZ*), measured in *WT* (blue) and two independent Δ*gcrA xylX*::P*xyl-gcrA* mutants (red and green), during 6 hours (h) of induction (xylose) of *gcrA* expression. Error (black bar) is shown as S.D. (n=3).

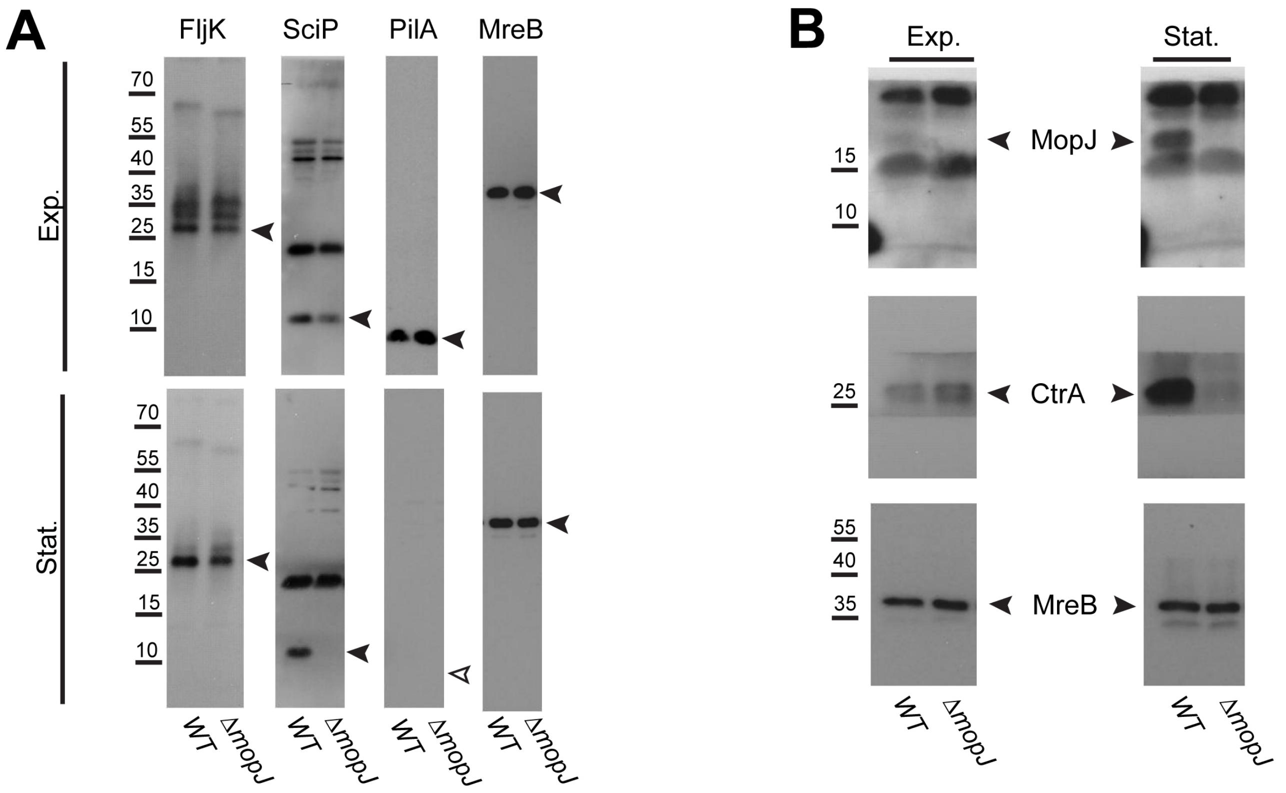

Supplementary Figure 4. Effect of Δ*mopJ* mutation on abundance of CtrA-dependent proteins and induction of MopJ in stationary phase. A. Immunoblot showing the steady-state levels of the major flagellin FljK, the SciP negative regulator and the PilA structural subunit of the pilus filament in *WT* and Δ*mopJ* cells in exponential (Exp.) and stationary (Stat.) phase. The steady-state level of the MreB actin are shown as a loading control (blots are also shown in Figure 2C). **B.** CtrA abundance is downregulated in exponential (Exp.) and stationary (Stat.) phase Δ*mopJ versus WT* cells. MreB steady-state levels are shown as loading control (see Figure 2C). **(A, B)** Molecular sizemarkers are indicated on the left. Filled arrowheads indicate the position of the proteins shown in Figure 2. The empty arrowhead denotes the absence of PilA in stationary phase versus exponential phase.

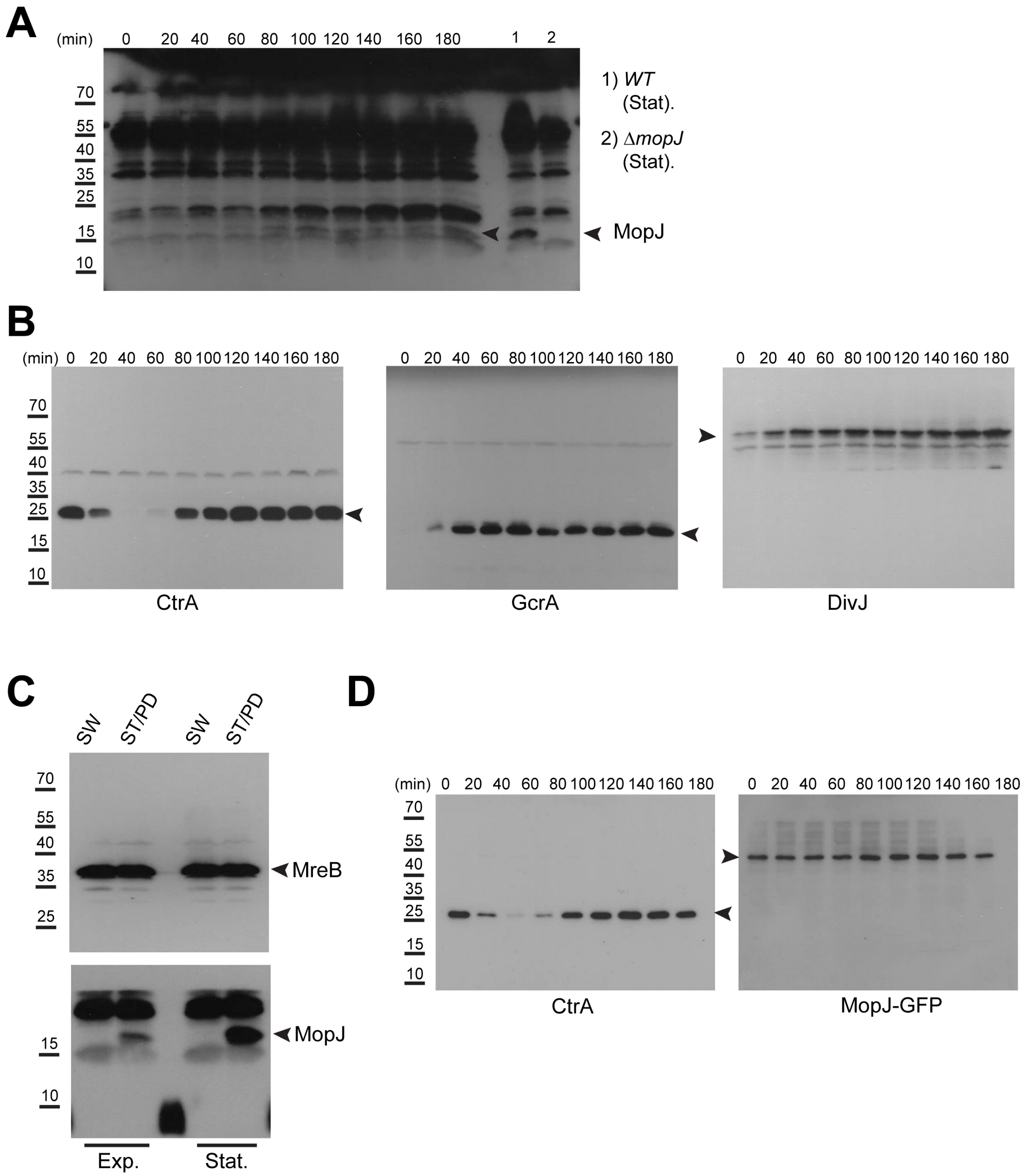

Supplementary Figure 5. Accumulation of MopJ during the stalked cell (S-) phase. (A, B) Immunoblots showing the steady-state levels of MopJ (**A**), CtrA, GcrA and DivJ (**B**) in sychronized NA1000 (*WT*) cells during exponential phase. Panel A also shows MopJ in stationary phase (Stat.) *WT* cells (1) and its absence in Δ*mopJ* cell (2) (blots also shown in Figure 3A). **C.** Accumulation of MopJ in stalked/pre-divisional (ST/PD) cells, but not on swarmer (SW) cells in (Exp.) and stationary (Stat.) phase. MreB steady-state levels are shown as loading control (also shown in Figure 3B). **D.** Immunoblots showing MopJ-GFP and CtrA levels in synchronized *mopJ::mopJ-GFP* cells (see also Figure 3I). (**A, B, D**) The numbers above the blot indicate the time after synchronization in minutes (min). (**A-D**) The numbers on the left indicate molecular size markers. arrowheads indicate the position of the proteins shown in Figure 3.

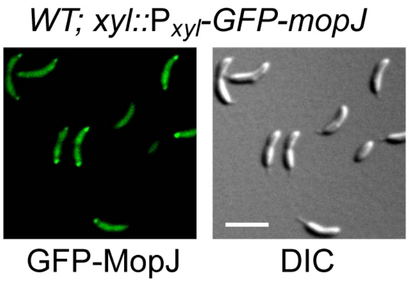

Supplementary Figure 6. Localization of GFP-MopJ. Live-cell imaging of NA1000 (*WT*) cells expressing GFP-MopJ from P_{xyl} at the *xylX* locus. Shown are fluorescence (left) and DIC (right) images. Bar 2 µm.

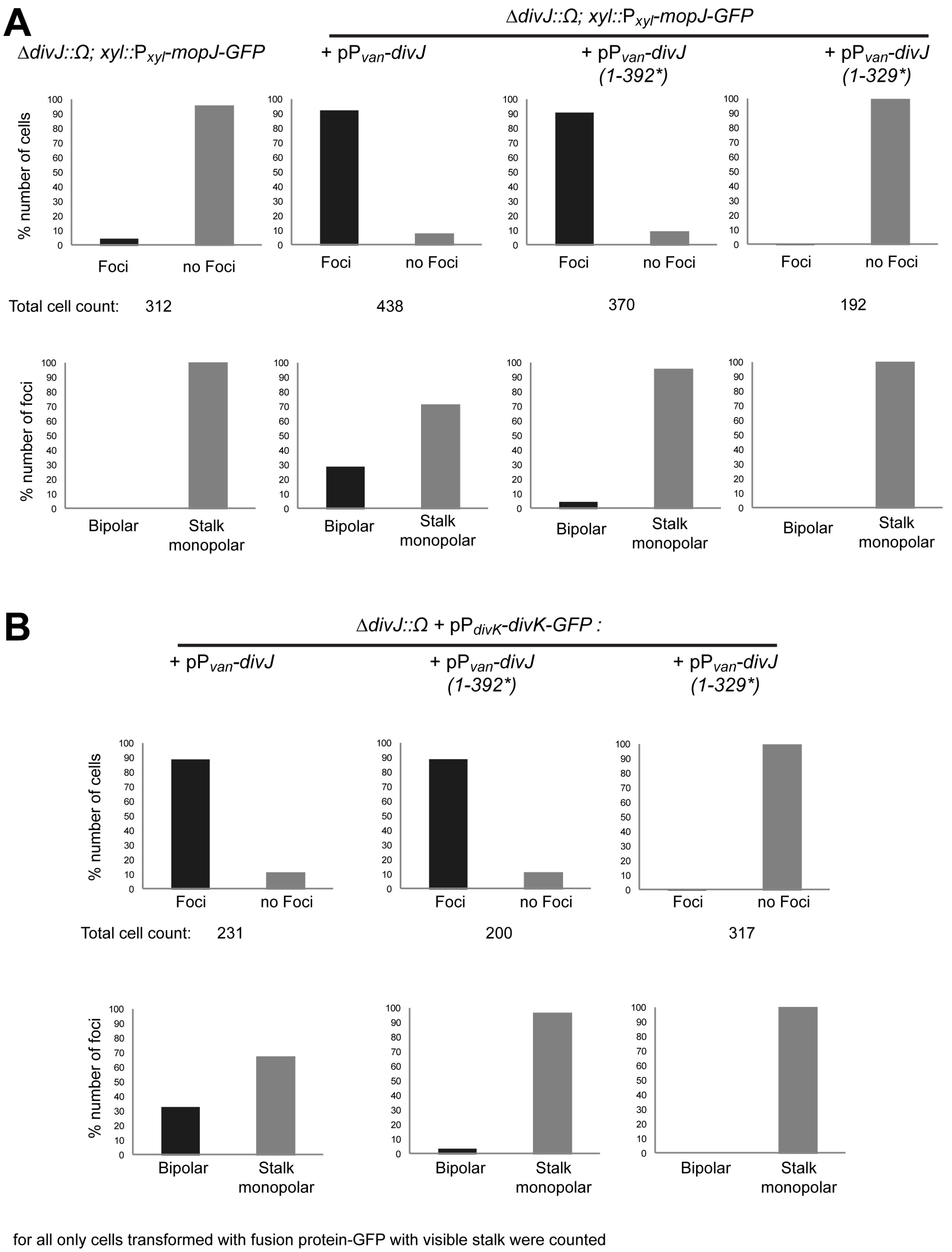

Supplementary Figure 7. Quantification of MopJ-GFP (A) and DivK-GFP foci (B). Various strains harboring P_{xyl}-*mopJ-GFP* at the *xylX* locus (A) or the pP_{divK}-*divK-GFP* plasmid (B) were grown in PYE supplemented with xylose (0.3%) and imaged by DIC and fluorescence microscopy. Foci were quantified.

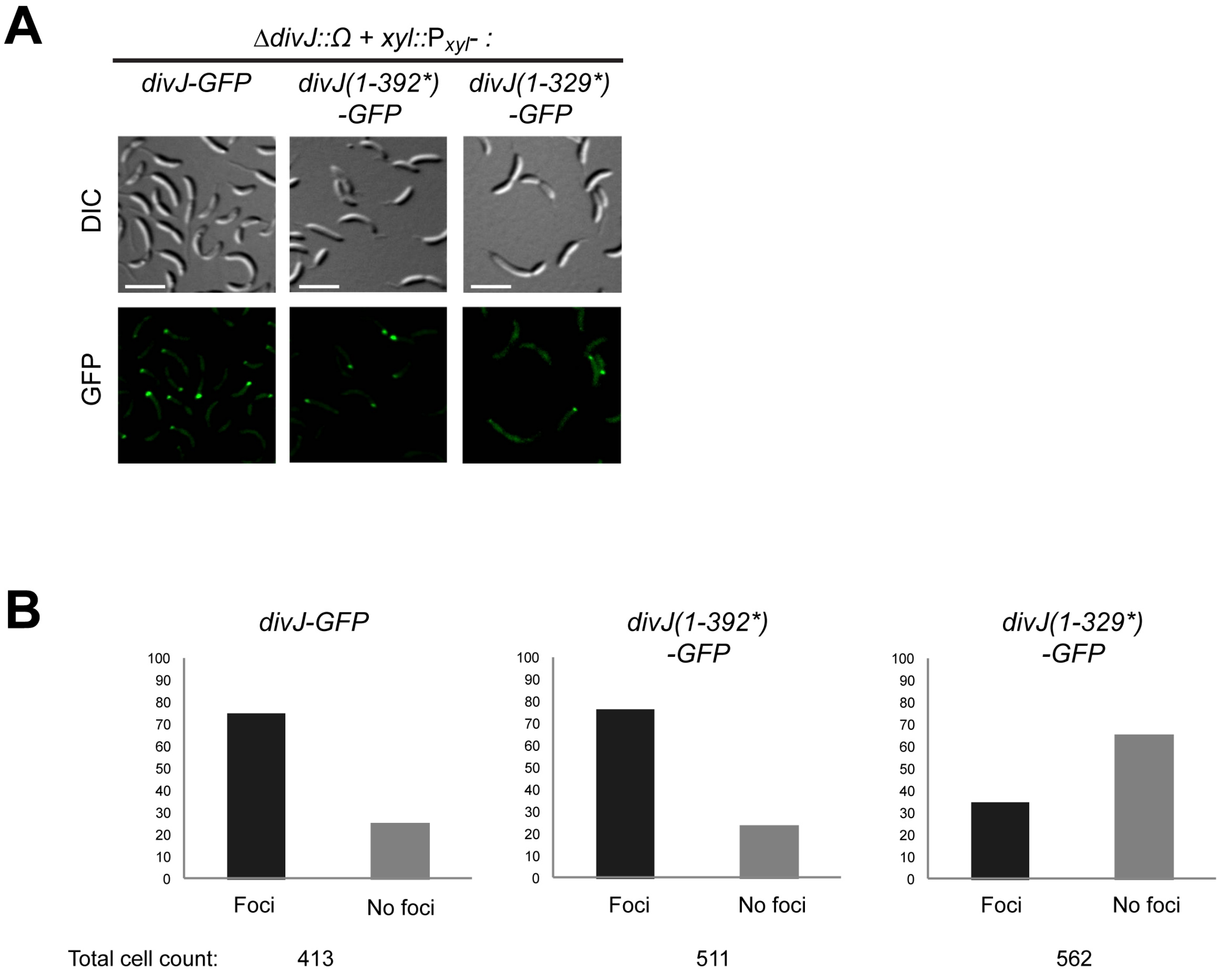

Supplementary Figure 8. Localization of DivJ derivatives. A. DivJ(*WT*), DivJ(1-392*) and DivJ(1-329*) fused to GFP were expressed at *xylX* locus in Δ*divJ* cells, grown in PYE supplemented with xylose (0.3%), and imaged by DIC and fluorescence microscopy. Bar, 2 µm **B.** Quantification of fluorescent foci.

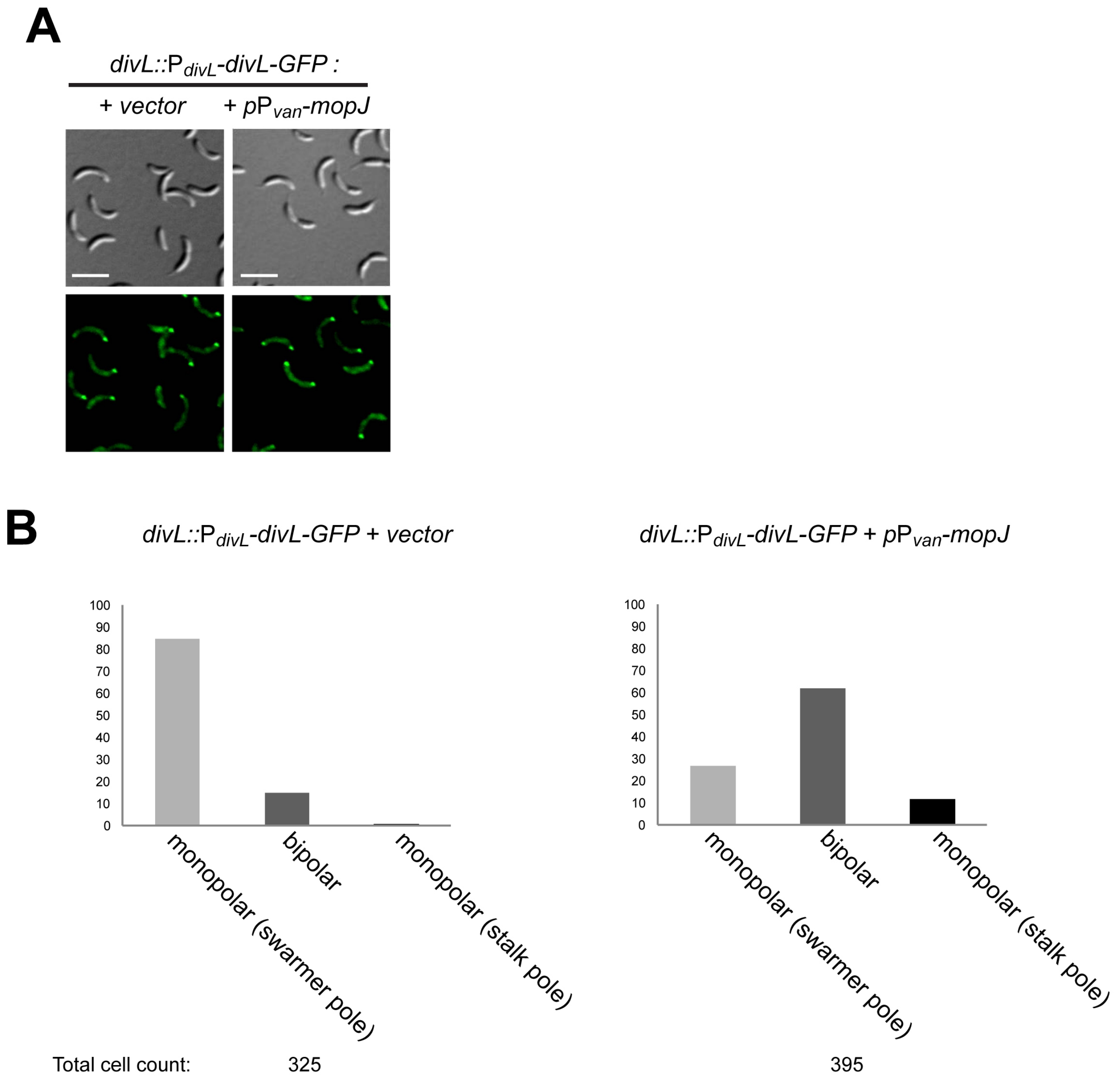

Supplementary Figure 9. Effect of MopJ overexpression on DivL-GFP localization. A. Effect of MopJ overexpression on DivL-GFP localization. The empty vector (pMT335) or the P_{van}-*mopJ* plasmid (pMT335-*mopJ)* was transformed into *divL*::P_{divL}-*divL-GFP* cells and the resulting cells imaged by DIC and fluorescence microscopy 4 hours after induction with vanillate (50 µM). Representatitive images are also shown in Figure 5F in the main text. Bar, 2 µm. **B.** Quantification of fluorescent foci.

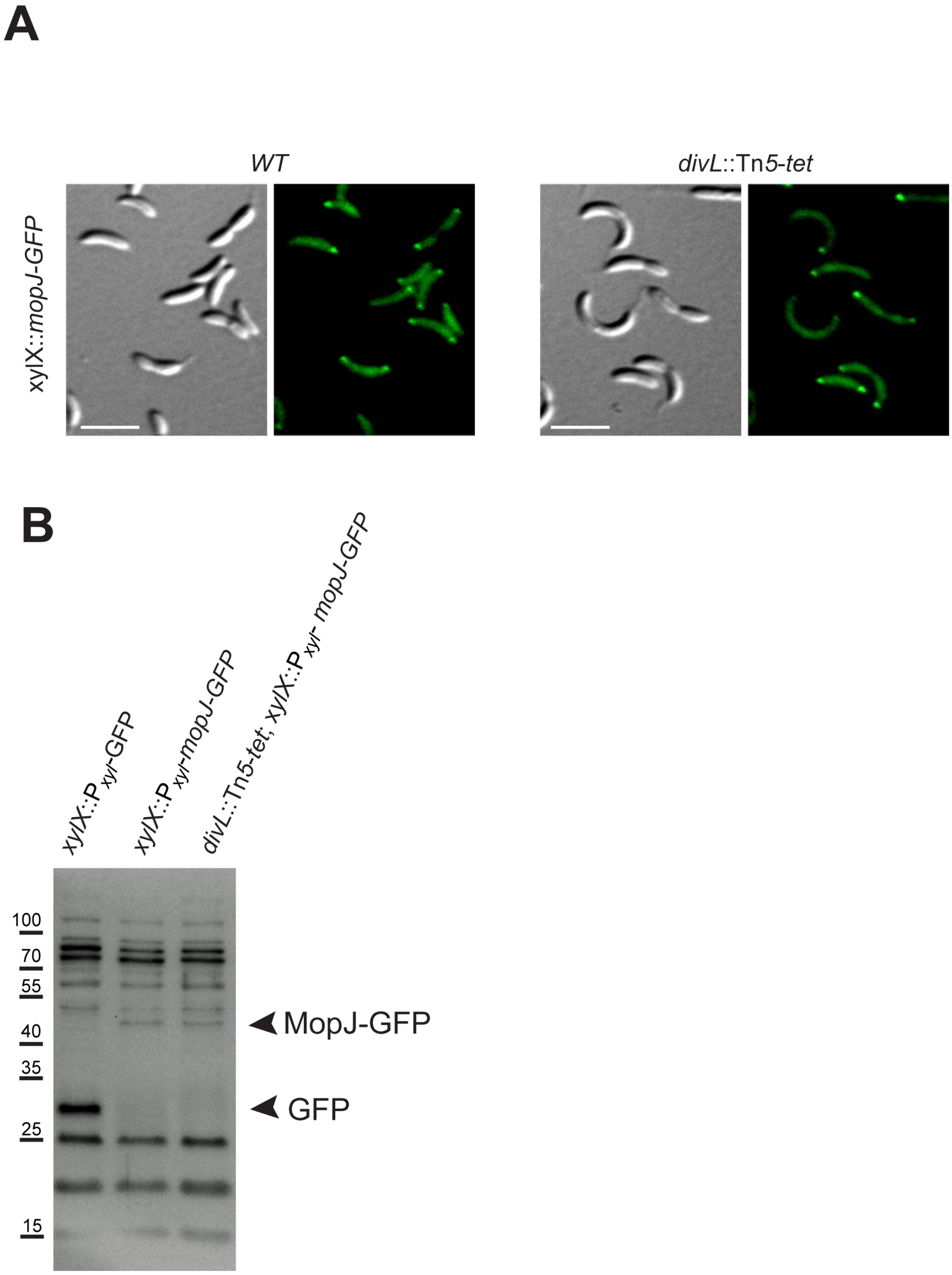

Supplementary Figure 10. MopJ-GFP is still localized a *divL*::Tn*5* mutant that does not express the HisKA domain. A. Localizaion of *divL*::Tn*5* cells harboring P_{xyl}-*mopJ-GFP* at the *xylX* locus were grown in PYE supplemented with xylose (0.3%) and imaged by DIC and fluorescence microscopy. Bar, 2 µm. **B.** Immunoblotting of extracts from cells shown in A as well as GFP-expressing control cells to detect MopJ-GFP or free GFP using antibodies to GFP.

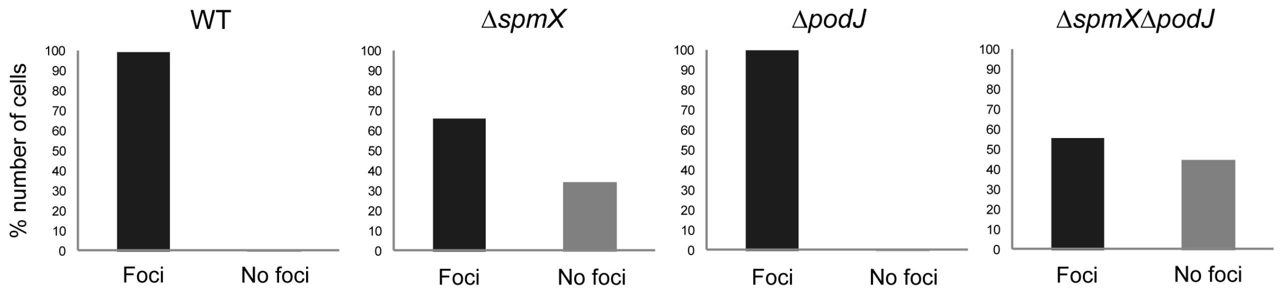

Total cell count: 310 249 268 137

only cells transformed with MopJ-GFP expression construct having visible stalks were counted

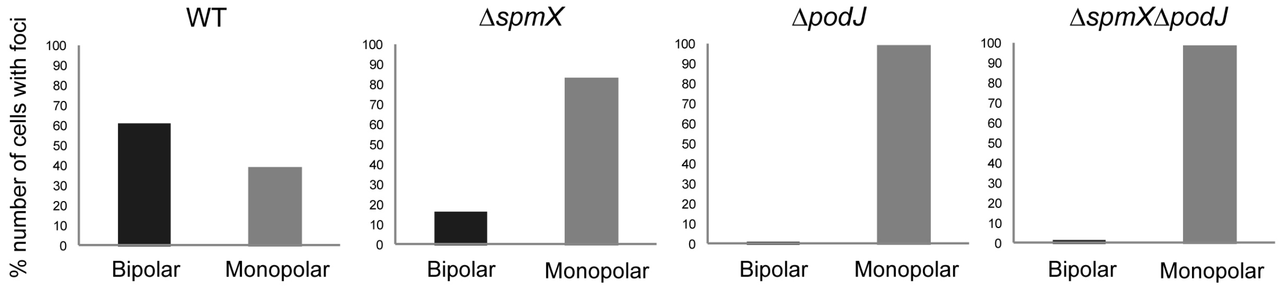

Monopolar means one focus at the stalked pole. Foci at the swarmer pole (not counted) are very rare.

Supplementary Figure 11. Quantfication of MopJ-GFP. Various strains harboring P_{xyl}-*mopJ-GFP* at the *xylX* locus were grown in PYE supplemented with xylose (0.3%) and imaged by DIC and fluorescence microscopy. Foci were quantified

Supplementary Tables

	Promoter region; exponential phase							
	P_{fljM}-*lacZ*		P_{sciP}-*lacZ*		P_{pilA}-*lacZ*		P_{fljK}-*lacZ*	
Strain	Value %	Difference	Value %	Difference	Value %	Difference	Value %	Difference
WT	100	–	100	–	100	–	100	–
Δ*mopJ*	75	-25	82	-18	86	-14	62	-38

Supplementary Table 1. Percentage values (value %) relative to absolute measurements of LacZ activity in exponential phase represented in Figure 2A. *WT* absolute value is referred as 100%. Percentage values indicating the difference between *WT* and Δ*mopJ* cells are also shown (downregulation is refered as minus, up-regulation is refered as plus).

	Promoter region; stationary phase							
	P_{fljM}-*lacZ*		P_{sciP}-*lacZ*		P_{pilA}-*lacZ*		P_{fljK}-*lacZ*	
Strain	Value %	Difference	Value %	Difference	Value %	Difference	Value %	Difference
WT	100	–	100	–	100	–	100	–
Δ*mopJ*	65	-35	59	-41	66	-34	52	-48

Supplementary Table 2. Percentage values (value %) relative to absolute measurements of LacZ activity in stationary phase represented in Figure 2B in the main text. *WT* absolute value is referred as 100%. Percentage values indicating the difference between *WT* and Δ*mopJ* cells are also shown (downregulation is refered as minus, up-regulation is refered as plus).

Supplementary Table 3. Bacterial strains used in this study.

Strains	Characteristics	Reference/source
Escherichia coli		
S17	RP4,Tc::Mu Km::Tn7	[1]
EC100D	*F- mcrA Δ(mrr-hsdRMS-mcrBC) Φ80dlacZΔM15 ΔlacX74 recA1 endA1 araD139 Δ(ara, leu)7697 galU galK λ- rpsL (StrR) nupG*	Epicentre
SS93	EC100D- pET28a-*mopJshort*	This work
Caulobacter crescentus		
NA1000	Synchronizable derivative of wild-type strain CB15	[2]
NR2642 (NS61)	NA1000 *mopJ::himar1* (nt 1082252)	This work
NR2652	NA1000 *xylX*::P_{xyl}-*mopJ-GFP*	This work
NR3915	NA1000 pMT374-P_{van}-*mopJ-Venus*	This work
SS1	NA1000 Δ*mopJ* A	This work
SS2	NA1000 Δ*mopJ* B	This work
SS11	NA1000 pMT335	This work
SS12	NA1000 Δ*mopJ* pMT335	This work
SS13	NA1000 Δ*mopJ* pMT335-*mopJ*	This work
SS18	NA1000 *mopJ*::P_{mopJ}-*mopJ-GFP*	This work
SS19	NA1000 Δ*divJ*::Ω *xylX*::P_{xyl}-*mopJ-GFP*	This work
SS20	NA1000 Δ*divJ*::Ω *xylX*::P_{xyl}-*mopJ-GFP* pMR20-P_{divJ}-*divJ*	This work
SS21	NA1000 Δ*divJ*::Ω *xylX*::P_{xyl}-*mopJ-GFP* pMT335	This work
SS22	NA1000 Δ*divJ*::Ω *xylX*::P_{xyl}-*mopJ-GFP* pMT335-*divJ*	This work
SS23	NA1000 Δ*divJ*::Ω *xylX*::P_{xyl}-*mopJ-GFP* pMT335-*divJ392*	This work
SS24	NA1000 Δ*divJ*::Ω *xylX*::P_{xyl}-*mopJ-GFP* pMT335-*divJ329*	This work
SS25	NA1000 Δ*popZ*::Ω *xylX*::P_{xyl}-*mopJ-GFP*	This work
SS26	NA1000 Δ*spmX xylX*::P_{xyl}-*mopJ-GFP*	This work
SS27	NA1000 Δ*podJ xylX*::P_{xyl}-*mopJ-GFP*	This work
SS29	NA1000 p*lacZ*290-P*mopJ*	This work
SS30	NA1000 Δ*gcrA*::Ω *xylX*::P_{xyl}-*gcrA* p*lacZ*290-P*mopJ*	This work
SS31	NA1000 Δ*socB* Δ*clpP*::Ω	This work
SS32	NA1000 Δ*socB* Δ*clpX*::Ω	This work
SS33	NA1000 *xylX::*P_{xyl}-*clpX**	This work
SS34	NA1000 *ctrA::ctrA-M2*	This work
SS35	NA1000 Δ*mopJ ctrA::ctrA-M2*	This work
SS37	NA1000 Δ*mopJ* Δ*cpdR*::*tet*	This work
SS39	NA1000 pMR10	This work
SS40	NA1000 Δ*mopJ* pMR10	This work
SS42	NA1000 pMR10-P_{xyl}-*divK*	This work
SS43	NA1000 Δ*mopJ* pMR10-P_{xyl}-*divK*	This work
SS45	NA1000 pMR10-*divK-GFP*	This work
SS46	NA1000 pMR10-*divK-GFP* pMT335	This work
SS47	NA1000 pMR10-*divK-GFP* pMT335-*mopJ*	This work
SS48	NA1000 *xylX*::P_{xyl}-*divK-CFP* pMT374-P_{van}*mopJ-venus*	This work
SS49	NA1000 Δ*divJ*::Ω pMR10-*divK-GFP*	This work
SS50	NA1000 Δ*divJ*::Ω pMR10-*divK-GFP* pMT335	This work
SS51	NA1000 Δ*divJ*::Ω pMR10-*divK-GFP* pMT335-*divJ*	This work
SS52	NA1000 Δ*divJ*::Ω pMR10-*divK-GFP* pMT335-*divJ392*	This work

SS53	NA1000 Δ*divJ*::Ω pMR10-*divK-GFP* pMT335-*divJ329*	This work
SS60	NA1000 p*lacZ*290-P*fljM*	This work
SS61	NA1000 Δ*mopJ* p*lacZ*290-P*fljM*	This work
SS68	NA1000 p*lacZ*290-P*fljK*	This work
SS69	NA1000 Δ*mopJ* p*lacZ*290-P*fljK*	This work
SS76	NA1000 p*lacZ*290-P*pilA*	This work
SS77	NA1000 Δ*mopJ* p*lacZ*290-P*pilA*	This work
SS84	NA1000 p*lacZ*290-P*sciP*	This work
SS85	NA1000 Δ*mopJ* p*lacZ*290-P*sciP*	This work
SS94	NA1000 Δ*divJ*::Ω *xylX*::P_{xyl}-*divJ-GFP*	This work
SS95	NA1000 Δ*divJ*::Ω *xylX*::P_{xyl}-*divJ392-GFP*	This work
SS96	NA1000 Δ*divJ*::Ω *xylX*::P_{xyl}-*divJ329-GFP*	This work
SS97	NA1000 Δ*mopJ divL*::P_{divL}-*divL-GFP*	This work
SS98	NA1000 *divL*::P_{divL}-*divL-GFP* pMT335	This work
SS99	NA1000 *divL*::P_{divL}-*divL-GFP* pMT335-*mopJ*	This work
SS100	NA1000 Δ*spmX* Δ*podJ*	This work
SS101	NA1000 *xylX*::pXTCYC4 p*lacZ*290-P*mopJ*	This work
SS102	NA1000 *xylX*::pXTCYC4-*relA'-FLAG* p*lacZ*290-P*mopJ*	This work
SS103	NA1000 *xylX*::pXTCYC4-*relA'(E335Q)-FLAG* p*lacZ*290-P*mopJ*	This work
SS104	NA1000 Δ*spmX* Δ*podJ xylX*::P_{xyl}-*mopJ-GFP*	This work
SS105	NA1000 Δ*spoT* p*lacZ*290-P*mopJ*	This work
SS106	NA1000 *xylX*::P_{xyl}-*GFP-mopJ*	This work
MB746	NA1000 *divL::Tn5 xylX*::P_{xyl}-*mopJ-GFP*	This work
YB3202	NA1000 Δ*divJ*::Ω	[3]
GB255	NA1000 Δ*popZ*::Ω	[4]
PV17	NA1000 Δ*podJ*	[5]
Δ*cpdR* (Δ*CC0744*)	NA1000 Δ*cpdR*::*tet*	[6]
LS3707	NA1000 Δ*gcrA*::Ω *xylX*::P_{xyl}-*gcrA*	[7]
CAC139	NA1000 Δ*socB*	[8]
UJ200	NA1000 Δ*clpX*::Ω *xylX::*P_{xyl}-*clpX*	[9]
CJ828	NA1000 *xylX*::P_{xyl}-*divK-CFP*	[10]
JC835	NA1000 *xylX*::pXTCYC4	[11]
JC820	NA1000 *xylX*::pXTCYC4-*relA'-FLAG*	[11]
JC1198	NA1000 *xylX*::pXTCYC4-*relA'(E335Q)-FLAG*	[11]
AOF2	NA1000 *divL*::P_{divL}-*divL-GFP*	[12]
FC769	NA1000 Δ*spoT*	[13]

Supplementary Table 4. Plasmids used in this study.

Plasmids	Characteristics	Reference/source
pHPV414	Non replicative vector in *C. crescentus* harboring the *himar1* transposon (Kan^R)	[14]
pNPTS138	Non replicative vector in *C. crescentus* containing the *sacB* gene (Kan^R)	M.R.K. Alley, Imperial College London (unpublished)
pCWR296	pNPTS138 allowing *mopJ* deletion (double recombination, sucrose counter selection)	This work
pMT335	High copy number vector containing the leaky P*van*	[15]

	inducible promoter (Gent^R)	
pMT335-*mopJ*	pMT335 containing *mopJ* coding sequence	This work
pMT335-*divJ*	pMT335 containing *divJ* coding sequence	This work
pMT335-*divJ392*	pMT335 containing *divJ392* coding sequence	This work
pMT335-*divJ329*	pMT335 containing *divJ329* coding sequence	This work
pXGFP4	Non replicative vector in *C. crescentus* containing *GFP* gene for expression of fusion genes with *GFP* at the 3' end under the control of P*xyl* inducible promoter after the integration at chromosomal *xylX* locus (Kan^R)	M.R.K. Alley, Imperial College London, (unpublished)
pXGFP4-C1	Non replicative vector in *C. crescentus* containing *GFP* gene for expression of fusion genes with *GFP* at the 5' end under the control of P*xyl* inducible promoter after the integration at chromosomal *xylX* locus (Kan^R)	M.R.K. Alley, Imperia College London (unpublished)
pCWR282	pXGFP4 containing *mopJ* coding sequence	This work
pXGFP4-*divJ*	pXGFP4 containing *divJ* coding sequence without stop codon	This work
pXGFP4-*divJ392*	pXGFP4 containing *divJ392* coding sequence	This work
pXGFP4-*divJ329*	pXGFP4 containing *divJ329* coding sequence	This work
pGFP4-*mopJ*	Non replicative vector in *C. crescentus* derived from pXGFP4 without *xylX* locus containing *mopJ* coding sequence and the upstream region of 640 bp for expression of *mopJGFP* fusion gene under the control of P*mopJ* after the integration at chromosomal original locus (Kan^R)	This work
pXGFP4-C1-*GFP-mopJ*	pXGFP4-C1 containing *mopJ* coding sequence	This work
pMR10	Low copy number vector (Kan^R)	R. Roberts and C. Mohr, Stanford University (unpublished)
pMR10-P_{xyl}-*divK*	pMR10 containing *divK* gene under the control of P*xyl*	D. Hung, Stanford University (unpublished)
pMR10-*divK-GFP*	pMR10 containing *divK-GFP* fusion gene	[16]
pMT374	Low copy number vector for expression of genes under the control of P*van* inducible promoter (Tet^R)	[15]
pMT374-*mopJ-YFP*	pMT374 containing *mopJYFP* fusion gene	This work
pMO88	pMR20 containing *clpX* gene with mutation in ATP binding site under the control of P*xyl*	[17]
p*ctrA-M2*	pJM21 (Kan^R vector for integrating C-terminal M2 fusions) harboring the last 450bp of *ctrA*	[18]
p*lacZ*290	Low copy number vector containing *lacZ* gene (used to create *lacZ* transcriptional fusion) (Tet^R)	[19]
p*lacZ*290-P*mopJ*	P*lacZ*290 containing *mopJ* promoter region (-228 to	This work

	+78 relative to the ATG)	
p*lacZ*290-P*fljM*	p*lacZ*290 containing *fljM* promoter region	[20]
p*lacZ*290-P*sciP*	p*lacZ*290 containing *sciP* promoter region	[20]
p*lacZ*290-P*fljK*	p*lacZ*290 containing *fljK* promoter region	[21]
p*lacZ*290-P*pilA*	p*lacZ*290 containing *pilA* promoter region	[22]
pET28a	High copy number vector for expression of genes under the control of T7 promoter	(Novagen, Madison WI)
pET28a-*mopJshort*	pET28a containing *mopJshort* sequence	This work
pXTCYC4	Non replicative vector in *C. crescentus* for expression of genes under the control of P*xyl* after the integration at chromosomal *xylX* locus ($Gent^R$)	[15]
pXTCYC4-*relA'-FLAG*	pXTCYC4 containing *relA'-FLAG*	[11]
pXTCYC4-*relA'(E335Q)-FLAG*	pXTCYC4 containing *relA'(E335Q)-FLAG*	[11]

Supplementary Table 5. Oligonucleotides used in this study.

Oligonucleotides	Sequences
delmopJ_1- EcoRI	5'-AAAAAAGAATTCGGCCTTGATCAAGCAGGTTCAC-3'
delmopJ_1-BamHI	5'-AAAAAAGGATCCCGCCGTGGCGGATGCGCTCA-3'
delmopJ_2-BamHI	5'-AAAAAAGGATCCGCCGCCGAGAGCGGCTCGGG-3'
delmopJ_2- HindIII	5'-AAAAAAGCTTAGAGATTCACGCCGCACCGCAC-3'
mopJ-NdeI	5'-AAAAAACATATGTCCGCGCAATTGAGCGCA -3'
mopJ-EcoRI	5'-AAAAAAGAATTCAGGCCGCCCGAATCCCCGA-3'
PmopJ-EcoRI	5'-AAAAAAGAATTCCAAACCTGCGCGCAAGGCGAAATA-3'
PmopJ-XbaI	5'-AAAAAATCTAGAACGGAGGGACGCCCAGTAGGC-3'
mopJshort-NdeI	5'- AAAAAACATATGCTGCCGACCGTCAGCCTGATCGA-3'
mopJ-NheI	5'-AAAAAAGCTAGCGGCGACGGGCGAGGCAT-3'
mopJfusion-BamHI	5'-AAAAAAGGATCCCGGCCGCCCGAATCCCCG-3'
mopJfusion-BglII	5'-AAAAAAAGATCTGTCTCCGCGCAATTGAGCGCA-3'
mopJtest_1-fw	5'-GCATTTCTAGCCATTTCCAGTAA-3'
mopJtest_3-rev	5'-ATCATCGTCACGCTCGGCG -3'
divJ-NdeI	5'-AAAAAACATATGATCCTCCCCACCGCGCTAAAA -3'
divJ-MunI	5'-AAAAAACAATTGTCAGCGCGGCGCAAAGGCGATGAC-3'
divJ392-MunI	5'- AAAAAACAATTGTCACTTGGACATGTCCAGCACGTCGTT-3'
divJ329-MunI	5'-AAAAAACAATTGTCAGCGGCCGGCGGCCAAGGCCT-3'
divJfusion-MunI	5'-AAAAAACAATTGGCGCGGCGCAAAGGCGATGAC-3'
divJ392fusion-MunI	5'-AAAAAACAATTGCTTGGACATGTCCAGCACGTCGTT-3'
divJ329fusion-MunI	5'-AAAAAACAATTGGCGGCCGGCGGCCAAGGCCT-3'
divJ_middle	5'-CGCTACGCCAGCGAGA-3'
divJ_midlle-rev	5'-CCAGCGCGGGGTTGAA-3'
PxylX	5'-AGGATTTCGCGCTGGTCAGACAA-3'
egfp-rev	5'-CAGGATGGGCACCACC-3'
pXGFP-fw	5'-GCGGTAGGCGTGTACG -3'
himar-Seq2	5'-GATATTGCTGAAGAGCTTGGCGGCGAA-3'
himar-TnSeq	5'-AGACCGGGGACTTATCAGCCAACCTGTTA-3'

References

1. Simon R, Priefer U, Puhler A. A broad host range mobilization system for in vivo genetic engineering: transposon mutagenesis in gram negative bacteria. *Nat Biotechnol* **1**, 784-790 (1983).

2. Evinger M, Agabian N. Envelope-associated nucleoid from *Caulobacter* crescentus stalked and swarmer cells. *J Bacteriol* **132**, 294-301 (1977).

3. Pierce DL, O'Donnol DS, Allen RC, Javens JW, Quardokus EM, Brun YV. Mutations in DivL and CckA rescue a *divJ* null mutant of *Caulobacter crescentus* by reducing the activity of CtrA. *J Bacteriol* **188**, 2473-2482 (2006).

4. Bowman GR*, et al.* A polymeric protein anchors the chromosomal origin/ParB complex at a bacterial cell pole. *Cell* **134**, 945-955 (2008).

5. Viollier PH, Sternheim N, Shapiro L. Identification of a localization factor for the polar positioning of bacterial structural and regulatory proteins. *Proceedings of the National Academy of Sciences* **99**, 13831-13836 (2002).

6. Skerker JM, Prasol MS, Perchuk BS, Biondi EG, Laub MT. Two-component signal transduction pathways regulating growth and cell cycle progression in a bacterium: a system-level analysis. *PLoS Biol* **3**, e334 (2005).

7. Holtzendorff J*, et al.* Oscillating global regulators control the genetic circuit driving a bacterial cell cycle. *Science* **304**, 983-987 (2004).

8. Aakre CD, Phung TN, Huang D, Laub MT. A bacterial toxin inhibits DNA replication elongation through a direct interaction with the beta sliding clamp. *Mol Cell* **52**, 617-628 (2013).

9. Jenal U, Fuchs T. An essential protease involved in bacterial cell-cycle control. *Embo J* **17**, 5658-5669 (1998).

10. Lam H, Matroule JY, Jacobs-Wagner C. The asymmetric spatial distribution of bacterial signal transduction proteins coordinates cell cycle events. *Dev Cell* **5**, 149-159 (2003).

11. Gonzalez D, Collier J. Effects of (p)ppGpp on the Progression of the Cell Cycle of *Caulobacter crescentus*. *J Bacteriol* **196**, 2514-2525 (2014).

12. Hughes HV, Huitema E, Pritchard S, Keiler KC, Brun YV, Viollier PH. Protein localization and dynamics within a bacterial organelle. *Proceedings of the National Academy of Sciences* **107**, 5599-5604 (2010).

13. Boutte CC, Crosson S. The complex logic of stringent response regulation in *Caulobacter crescentus*: starvation signalling in an oligotrophic environment. *Molecular Microbiology* **80**, 695-714 (2011).

14. Viollier PH*, et al.* Rapid and sequential movement of individual chromosomal loci to specific subcellular locations during bacterial DNA replication. *PNAS* **101**, 9257-9262 (2004).

15. Thanbichler M, Iniesta AA, Shapiro L. A comprehensive set of plasmids for vanillate- and xylose-inducible gene expression in *Caulobacter crescentus*. *Nucleic Acids Research* **35**, e137 (2007).

16. Jacobs C, Hung D, Shapiro L. Dynamic localization of a cytoplasmic signal transduction response regulator controls morphogenesis during the *Caulobacter* cell cycle. *Proc Natl Acad Sci U S A* **98**, 4095-4100 (2001).

17. Potocka I, Thein M, M OS, Jenal U, Alley MR. Degradation of a *Caulobacter* soluble cytoplasmic chemoreceptor is ClpX dependent. *J Bacteriol* **184**, 6635-6641 (2002).

18. Domian IJ, Quon KC, Shapiro L. Cell type-specific phosphorylation and proteolysis of a transcriptional regulator controls the G1-to-S transition in a bacterial cell cycle. *Cell* **90**, 415-424 (1997).

19. Gober JW, Shapiro L. A developmentally regulated *Caulobacter* flagellar promoter is activated by 3' enhancer and IHF binding elements. *Mol Biol Cell* **3**, 913-926 (1992).

20. Fumeaux C*, et al.* Cell cycle transition from S-phase to G1 in *Caulobacter* is mediated by ancestral virulence regulators. *Nature communications* **5**, 4081 (2014).

21. Wingrove JA, Mangan EK, Gober JW. Spatial and temporal phosphorylation of a transcriptional activator regulates pole-specific gene expression in *Caulobacter*. *Genes Dev* **7**, 1979-1992 (1993).

22. Skerker JM, Shapiro L. Identification and cell cycle control of a novel pilus system in *Caulobacter crescentus*. *Embo J* **19**, 3223-3234 (2000).
